# Genetic diversity and signatures of selection in four indigenous horse breeds of Iran

**DOI:** 10.1038/s41437-023-00624-7

**Published:** 2023-06-12

**Authors:** Seyedeh Fatemeh Mousavi, Mohammad Razmkabir, Jalal Rostamzadeh, Hamid-Reza Seyedabadi, Rakan Naboulsi, Jessica L. Petersen, Gabriella Lindgren

**Affiliations:** 1 Department of Animal Science, Faculty of Agriculture, University of Kurdistan, Sanandaj, Iran; 2 Department of Animal Breeding and Genetics, Swedish University of Agricultural Sciences, Uppsala, Sweden; 3 Animal Science Research Institute of Iran, Agricultural Research Education and Extension Organization (AREEO), Karaj, Iran; 4 Childhood Cancer Research Unit, Department of Women’s and Children’s Health, Karolinska Institute, Tomtebodavägen 18A, 17177 Stockholm, Sweden; 5 Department of Animal Science, University of Nebraska, Lincoln, NE USA; 6 Center for Animal Breeding and Genetics, Department of Biosystems, KU Leuven, 3001 Leuven, Belgium

**Keywords:** Animal breeding, Evolutionary biology

## Abstract

Indigenous Iranian horse breeds were evolutionarily affected by natural and artificial selection in distinct phylogeographic clades, which shaped their genomes in several unique ways. The aims of this study were to evaluate the genetic diversity and genomewide selection signatures in four indigenous Iranian horse breeds. We evaluated 169 horses from Caspian (*n* = 21), Turkmen (*n* = 29), Kurdish (*n* = 67), and Persian Arabian (*n* = 52) populations, using genomewide genotyping data. The contemporary effective population sizes were 59, 98, 102, and 113 for Turkmen, Caspian, Persian Arabian, and Kurdish breeds, respectively. By analysis of the population genetic structure, we classified the north breeds (Caspian and Turkmen) and west/southwest breeds (Persian Arabian and Kurdish) into two phylogeographic clades reflecting their geographic origin. Using the de-correlated composite of multiple selection signal statistics based on pairwise comparisons, we detected a different number of significant SNPs under putative selection from 13 to 28 for the six pairwise comparisons (FDR < 0.05). The identified SNPs under putative selection coincided with genes previously associated with known QTLs for morphological, adaptation, and fitness traits. Our results showed *HMGA2* and *LLPH* as strong candidate genes for height variation between Caspian horses with a small size and the other studied breeds with a medium size. Using the results of studies on human height retrieved from the GWAS catalog, we suggested 38 new putative candidate genes under selection. These results provide a genomewide map of selection signatures in the studied breeds, which represent valuable information for formulating genetic conservation and improved breeding strategies for the breeds.

## Introduction

Different agro-ecological conditions across Iran have resulted in the development of distinct indigenous horse breeds. Iran has five officially registered horse breeds, including the Persian Arabian, Caspian, Turkmen (Akhal-Teke), Dareshuri, and Kurdish, with long-standing husbandry, cultural, and historical importance (Fotovati 2000). Unfortunately, these breeds are classified as endangered populations (Sadeghi et al. 2019) due to their small population size and uncontrolled admixture with exotic breeds. Due to the lack of active and strong equine breed associations in Iran, there is no clear strategy for the breeding of these populations. Breed purity is the most important factor for the pricing of Caspian and Kurdish horses and, as a result, horse owners mate their mares with stallions that have a valid pedigree. In Kurdish and Turkmen horses, due to the availability of breed and beauty competitions, in addition to their pedigree information, stallions are selected and mated based on their own and their relatives’ performance in these competitions. Since genetic diversity is crucial for a population to adapt to changing environments, an assessment of genetic diversity and the selection pressures behind it will help to choose the most appropriate solution in this situation. Consequently, breeding programs must be designed to prevent further loss of genetic diversity.

The Arabian horse is distributed worldwide, with a population size of >1 million (World Arabian Horse Organization, WAHO). However, this breed is divided into relatively small breeding populations in many countries in the Middle East, including Egypt, Saudi Arabia, Syria, and Iran, each with very different phenotypic attributes (Forbis 1976). Based on the current evidence from Y-chromosome analysis, three discrete Y-chromosomal haplotypes specific to the Arabian horses were detected (Remer et al. 2022). The Turkmen horse is thought to be the oldest surviving horse breed in the world, divided into several populations with unique genetic resources, which mostly differ in type, conformation, and usage (Jiskrová et al. 2016). The Caspian horse is a small ancient breed, with its natural habitat in the north of Iran. Although it was suggested that the Turkmen and Caspian horses might be ancestral to all forms of the oriental horse (Firouz 1998), most modern male lineages were derived from two major subclades, including Turkmen and Arabian lineages based on Y-chromosome analysis (Wallner et al. 2017). The study by Remer et al. (2022) showed a clear distinct Y haplotype phylogeny between Turkmen and Arabian horse breeds. The Kurdish horse originates from the west of Iran, an area characterized by mountainous topography and a moderately cold climate. This environment created a unique horse population resistant to harsh environmental conditions. The Iranian Kurdish horse has a small population size of only about 7000 horses. Therefore, an optimized selection strategy and mating program has been suggested to maintain genetic diversity (Nazari et al. 2022). Despite several studies on different native Iranian horse breeds that assessed the genetic diversity, parentage verification, and genetic structure of the populations using microsatellite or single-nucleotide polymorphism (SNP) (Amjadi et al. 2021; Nazari et al. 2022; Sadeghi et al. 2019; Salek Ardestani et al. 2022; Seyedabadi et al. 2006; Shahsavarani and Rahimi-Mianji 2010), the majority of the studies were performed on a single breed (Ala-Amjadi et al. 2017; Gharahveysi and Irani 2011; Nazari et al. 2022; Rahimi-Mianji et al. 2015; Seyedabadi et al. 2006). However, Moridi et al. (2013) studied the mitochondrial DNA (mtDNA) diversity and origin of five Iranian native horses, including Turkmen, Kurdish, Caspian, Iranian Arabian, and Sistani breeds. In 2019, Sadeghi et al. (2019) performed a study using five Iranian horse breeds, including Turkmen, Kurdish, Caspian, Iranian Arabian, and Dareshuri, where they focused on the genetic diversity and genomewide selection signatures in the Persian Arabian. Subsequently, Yousefi-Mashouf et al. (2021) compared the Kurdish horse population with the Persian Arabian and American Thoroughbred populations. Their results showed a significant population structure pattern between Kurdish and Persian Arabian samples. To date, there have been no genomewide SNP-based studies of genetic diversity and on detecting signatures of selection in multiple Iranian native breeds.

Different statistical tests may not generate consistent results for detecting signatures of selection due to variation in their power as a consequence of demographic history, type of selection, genetic architecture, and experimental design, as well as variation in sensitivity to sampling design (Lotterhos and Whitlock 2015; Schlamp et al. 2016; Vatsiou et al. 2016). ***Combining the results from multiple tests generally perform better with more distinguishable footprints of positive selection, and importantly in a closer proximity to the real selected locus (Lotterhos et al. 2017b). Many strategies have been proposed to overcome these issues, such as methods based on combining P-values of different test statistics (composite measures of selection) (Lotterhos and Whitlock 2015; Randhawa et al. 2014). To improve statistical power and resolution, we combined multiple statistics of signatures of selection including *F*_*ST*_ (Wright 1949), FLK (the extension of the Lewontin and Krakauer test) (Bonhomme et al. 2010) and xp-EHH (cross-population extended haplotype homozygosity) (Sabeti et al. 2007) within a single de-correlated composite of multiple selection signals (DCMS) framework (Ma et al. 2015). This calculation combines *P*-values while considering the correlation between the various statistics.

This study evaluates four Iranian horse breeds: Turkmen, Caspian, Kurdish, and Persian Arabian. We used the genomewide SNP information in a comprehensive genomewide analysis of the genetic diversity of Iranian horses to investigate the population structure and their genetic differences. Additionally, we applied a genomewide analysis to detect breed-specific genomic regions that display signals characteristic of selection. The selected genomic regions may contain variants important for the unique physiological traits and adaptations characteristics of each Iranian horse breed.

## Materials and methods

### Samples and populations

The data represent four different data sets. In this study, we used a data set collected by the Animal Science Research Institute of IRAN (ASRI), with the main focus on establishing genomic information for the Kurdish breed (*n* = 81). Horses in the ASRI data set were selected based on pedigree information, without crossbreeding in their pedigree, and those passed microsatellite parentage testing. These samples were collected from four Iranian provinces (Kermanshah, Kurdistan, Isfahan, and Kerman). One Persian Arabian (collected from Tehran) and two Turkmen (collected from Isfahan and Tehran) samples were present in the ASRI data set used in this study. A total of 86 samples from three breeds, including Turkmen (*n* = 34), Caspian (*n* = 22), and Kurdish (*n* = 30), were collected by the National Animal Breeding Center (ABC) from six provinces (Kermanshah, Kurdistan, Isfahan, Golestan, Mazandaran, and Tehran). This data set was collected between 2014 and 2015. For each breed, horses were sampled based on their pedigree. To extend and include Persian Arabian horses in our samples, we merged our data set with the data from two studies: 96 samples from Sadeghi et al. (2019), including Persian Arabian, Turkmen, Caspian, and Kurdish breeds, and 9 Persian Arabian samples from Cosgrove et al. (2020).

### Genotyping and quality control

Genomic DNA from the ASRI and ABC samples was extracted from more than 50 hair roots collected from the horse’s mane of each sample using the DNeasy Blood and Tissue kit (Qiagen, Germany). Then, samples were genotyped at Neogen GeneSeek Inc. (Lincoln, NE, USA) using the GGP Equine 70K SNP BeadChip array (Neogen GeneSeek, Lincoln, NE). Samples from the two other data sets were genotyped with the 670K Equine SNP chip as described previously (Cosgrove et al. 2020; Sadeghi et al. 2019). The annotation of SNP chromosomal positions was initially reported as EquCab2.0, and then converted to the most recently updated map using the lift genome annotations tool (https://genome.ucsc.edu/cgi-bin/hgLiftOver) based on the EquCab3.0 assembly (Kalbfleisch et al. 2018).

**Fig. 1:**
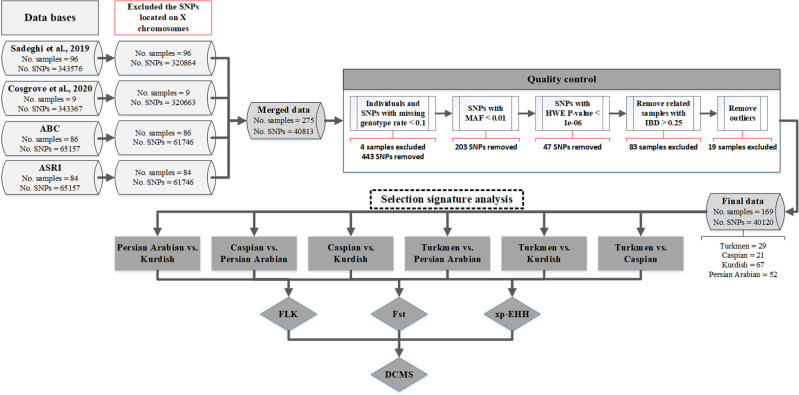
A flow chart analysis. Data preparation and identification of selection signatures in pairwise comparisons in Iranian horse breeds using different data sets.

The genotype data from different data sets were merged, keeping the loci that overlapped between the chips using PLINK V1.9 (Purcell et al. 2007). As our analysis focused on autosomal variants, we excluded the SNPs on sex chromosomes. After merging the data, we had 275 samples and 40,813 SNPs for further quality control (QC) (Fig. 1). The QC was performed using PLINK V1.9 (Purcell et al. 2007). First, SNPs and samples were filtered based on a less than 90% genotyping rate. Second, SNPs with a minor allele frequency (MAF) lower than 1% or those that departed from Hardy–Weinberg proportions at *P* < 10^–6^ were discarded. Third, as closely related individuals within each breed of the horse samples may provide biased sets for use in some of the analyses, we filtered individuals based on pairwise identity-by-descent (IBD) less than 0.25. The --genome option in PLINK was implemented to calculate the IBD values. The number of removed SNPs and samples after each step of QC are shown in Fig. 1. Before removing related individuals, Beagle V5.2 software was used to impute the sporadic missing genotypes in each population separately (Browning and Browning 2007). As well described by Pook et al. (2020), the effective population size (*N*_*e*_) can have a major effect on the error rate for imputation in Beagle, wherein the Markov chain uses the genetic data set to initialize a haplotype cluster and then initializes the Hidden Markov Model by identifying the most likely path through the haplotype cluster. Based on the default setting, this works well for imputation in outbred human populations, but the algorithm can be adjusted to the specific genetic structure of the respective data set by changing the *N*_*e*_ parameter. Pedigrees in Iranian horse breeds have much less genetic diversity than in human populations, which results in a smaller *N*_*e*_. To deal with this, we predicted the *N*_*e*_ for each breed using NeEstimator V2.1 (Do et al. 2014); the estimated *N*_*e*_ was used as the input for Beagle (see the section “Estimation of effective population size using genomewide SNP data”).

As LD-based pruning reduces the effect of ascertainment biases on diversity estimates (Malomane et al. 2018), a subset of SNPs was selected by linkage disequilibrium (LD)-based SNP pruning using PLINK for genetic diversity and population structure analysis. For principal component analysis (PCA), we applied the indep-pairwise command with an SNP window size of 50, 5 SNPs shifted per step, and the squared correlation between SNPs (*r*^2^) ≤ 0.5. For admixture and relatedness analysis, SNPs were pruned using the same sliding window and SNPs shifted per step, while the *r*^2^ threshold was 0.3. After LD-based SNP pruning, 28,387 and 22,523 SNPs remained for PCA, and admixture and relatedness analysis, respectively.

### Genetic distances, pairwise F_ST_, and absolute allele frequency difference (AFD)

The Cavalli-Sforza and Edwards Chord distance (Takezaki and Nei 1996) was calculated from the SNP information using the R package hierfstat (Goudet 2005). We applied the R package hierfstat to calculate the pairwise *F*_*ST*_ between the four main Iranian horse breeds (Goudet 2005). A bootstrap confidence interval was calculated for each pairwise *F*_*ST*_ comparison. As calculations of *F*_*ST*_ can be impacted by asymmetry in sample sizes, we calculated the absolute allele frequency difference (AFD) estimates to display the magnitude of differentiation (Berner 2019). The AFD at each locus was calculated as

$$AFD = \frac{{\mathop {\sum}\nolimits_{i = 1}^n {\left( {p_{i1} - p_{i2}|} \right.} }}{2},$$

where *n* is the number of alleles observed at the SNP, and *p*_i1_ and *p*_i2_ are the frequencies of allele *i* in populations 1 and 2, respectively.

### Estimation of effective population size using genomewide SNP data

We estimated both historical and contemporary *N*_*e*_ for all breeds using all 40,120 quality-controlled SNPs. SNeP software was implemented to estimate the historical *N*_*e*_ based on LD information (Barbato et al. 2015). We used the default options with a maximum distance of 4000 kbps.

The contemporary *N*_*e*_ was calculated using NeEstimator V2.1 (Do et al. 2014). We used a single-sample estimator based on LD information that implements a bias correction when the sample size is less than the true effective size (Waples and Do 2008). A random mating population was assumed for the analysis. MAFs of 0.1, 0.05, 0.02, and 0 for all SNPs were included in the analysis to screen out the effect of rare alleles on *N*_*e*_ estimation. The jackknife method was used to calculate the confidence intervals, as this method can perform better than parametric confidence intervals for the LD method (Waples and Do 2008). To omit comparisons of loci on the same chromosome, LD was calculated among pairs of SNPs located on different chromosomes.

### Genetic diversity and population structure

First, we performed a PCA on the LD pruned subset of SNPs using the “prcomp” function (with scale = TRUE) of the R statistical software (R Core Team 2013). Then, we used the first three PCs to identify and remove outlier samples, as possibly there are pedigree errors, especially in the past if a microsatellite test was not used for a pedigree check, leading to unreported crossbred individuals. The local outlier factor (LOF) algorithm was implemented to score each data point in a multidimensional process. LOF is an unsupervised anomaly detection method that computes the local density deviation of a given data point when only a restricted neighborhood of each object is taken into account (Breunig et al. 2000). We assumed that the outliers have a substantially lower density than their neighbors, which results in a gap from the rest of the data. All detected outliers were excluded in subsequent analyses. After removing outliers, PCA was conducted again on an LD pruned subset of SNPs without outliers to investigate the relationships among populations.

We also evaluated the relationship among the populations using the R package SNPRelate (Zheng et al. 2012). First, we calculated the individual dissimilarities for each pair of individuals using the “snpgdsDiss” function. Second, a hierarchical cluster analysis on this dissimilarity matrix was performed using the “snpgdsHCluster” function. Finally, subgroups of individuals were determined via a specified dendrogram from this hierarchical cluster analysis using the “snpgdsCutTree” function with 50,000 permutations.

We applied the software ADMIXTURE in order to infer breed origins and quantify the admixture of the populations (Alexander et al. 2009). For each individual, ancestry proportions were estimated with a priori-defined ancestry components (K) as a presumption of the number of ancestral populations (Alexander and Lange 2011). We implemented a 5-fold cross-validation for each K (ranged from 1 to 7) to estimate the standard error values for comparison, as the lowest one is the most likely number of ancestral populations.

LD between all SNPs was calculated with the --r2 command in PLINK and mean LD was expressed as a function of distance to determine the diminishing of LD with increasing physical distance between SNPs.

### Runs of homozygosity (ROH)

ROH, the indicator of genomic autozygosity due to a long stretch of homozygous SNPs that may have been inherited from a recent common ancestor, were studied to increase our understanding of the genetic diversity status of the four native horse populations. ROH segments along the genome were calculated based on the sliding window method using the R package detectRUNS (Biscarini et al. 2018). To estimate the ROH in four Iranian horse breeds, we used all common 40,120 quality-controlled SNPs in each breed separately. The following options were used: the sliding window size (windowSize) = 15, minimum number of homozygous SNPs in a run (minSNP) = 20, the threshold of overlapping windows (threshold) = 0.05, minimum number of SNP per kbp (minDensity) = 1/168, maximum distance between two SNPs (maxGap) = 10^6 ^bps, and the minimum length of a homozygous run (minLengthBps) = 500 kbps. The detected ROHs were divided into five categories, including 0 to < 4 Mb, 4 to < 8 Mb, 8 to < 16 Mb, 16 to < 32 Mb, and > 32 Mb. For each of the ROH length categories, the number of runs in each breed was calculated by averaging the sum of all ROHs per animal within the breeds. To identify the position of a ROH peak, we used a threshold of 0.7, which indicates the ROH shared in more than 70% of the individuals within each population. The genomic inbreeding coefficient using ROH information (*F*_*ROH*_) was calculated as follows:

$$F_{ROH} = \frac{{\mathop {\sum}\nolimits_{j = 1}^p {L_{ROHj}} }}{{L_{genome}}}$$

where *L*_*ROHj*_ is the length of the *j*th (*j* = 1,…, *p*) ROH detected in an individual, and *L*_genome_ is the total length of the genome that was used.

### Selection sweep

We performed six pairwise comparisons, including (i) Turkmen vs. Caspian, (ii) Turkmen vs. Kurdish, (iii) Turkmen vs. Persian Arabian, (iv) Caspian vs. Kurdish, (v) Caspian vs. Persian Arabian, and (vi) Persian Arabian vs. Kurdish, to determine the genetic regions under selection using *F*_*ST*_ (Wright 1949), FLK (Bonhomme et al. 2010), and xp-EHH (Sabeti et al. 2007) analyses.

The *F*_*ST*_ statistic was estimated using the program VCFtools v0.1.16 (Danecek et al. 2011). For each comparison, Z transformation of the mean of *F*_*ST*_ values (Z(*F*_*ST*_)) was computed for all SNPs using the scale function in R software.

FLK is an extension of the original LK statistic (Lewontin and Krakauer 1973) that is based on the variance of *F*_*ST*_ on comparing the observed and expected variances of *F*_*ST*_ across loci. FLK accounts for complex demographic structures and differences in the effective population size using a phylogenetic estimation of the population’s kinship (F) matrix to consider historical branching and heterogeneity of the genetic drift (Bonhomme et al. 2010). The FLK’s *P* values were computed using the hapFLK software (Fariello et al. 2013). In this analysis, the kinship matrix was calculated from the Reynolds’ genetic distances between populations (Reynolds et al. 1983) and a phylogenetic tree was fitted from these distances using the neighbor-joining algorithm. For each comparison, a negative log *P*-value was used.

An LD-based method, EHH, proposed by Sabeti et al. (2007) is useful in the detection of long homozygous regions that are under partial or soft selective sweep within a population. This test can be extended for implementation in the cross-population aspect, xp-EHH, which compares populations regarding their haplotypes (Sabeti et al. 2007). The xp-EHH scores were calculated using the REHH package (Gautier and Vitalis 2012) in R to determine selected mutations with a higher frequency than expected according to their haplotype length.

### De-correlated composite of multiple signals (DCMS)

Combining various test statistics of the selection signature improves the signal-to-noise ratio and increases the resolution to identify selected genomic regions (Lotterhos et al. 2017a; Ma et al. 2015). We combined three genomewide selection signature statistics into a single DCMS value (Ma et al. 2015). DCMS accounts for the correlation between the different statistics while combining *P*-values produced by several statistics for each locus into a single DCMS measurement.

We calculated the genomewide DCMS for each pairwise comparison by combining the aforementioned statistics (*F*_*ST*_, FLK, and xp-EHH) for each locus as described in Yurchenko et al. (2018). The genomewide *P*-values were converted to fractional ranks for each statistic using the stat_to_pvalue function represented in the R package MINOTAUR (Verity et al. 2017) with the right-tailed test for all the three methods. The covariance matrix between the different statistics was estimated using the CovNAMcd function from the rrcovNA package in R (Todorov et al. 2011) with alpha = 0.75 and 30,000 randomly sampled SNPs to calculate an *n* × *n* covariance matrix. Then, this calculated covariance matrix was used as an input for the DCMS function in the MINOTAUR R package to calculate the DCMS statistics. The output of the DCMS function, i.e. the DCMS statistics, was fitted to the normal distribution using the robust linear model method implemented in the rlm R function (model = rlm (dcms ~ 1), where the dcms is a vector of the raw DCMS values) of the MASS package (Venables and Ripley 2013). The rlm R function produced a mean and standard deviation that were used as inputs in the pnorm R function with a lower.tail attribute equal to FALSE. Finally, the DCMS *P* values were transformed into the corresponding *q*-values based on the Benjamini and Hochberg procedure (Benjamini and Hochberg 1995) to control the multiple testing false discovery rate (FDR < 0.05). This transformation was conducted using the p.adjust R function.

### Identification of candidate genes, QTLs, and functional analysis

Equine gene annotations and gene ontology (GO) information from the horse genome assembly build Equcab3.0 were downloaded from Biomart in November 2021 (Kasprzyk 2011). To identify the putative candidate genes under selection, we extracted genes including SNPs with a *q*-value < 0.05 in the coding region (both exon and intron) or including ± 40 kbps at both sides to control the regulatory regions. We selected 40 kbps extension based on the LD decay result from all breeds, where the average *r*^2^ was >0.2 (see the section “Population genetic structure and linkage disequilibrium”). The gene ontology (GO) pathway enrichment analysis was performed by the enricher function from the clusterProfiler R package (Wu et al. 2021). A list of significant quantitative trait loci (QTLs) for the horse database (horse QTLdb) was retrieved from animalQTLdb (Hu et al. 2007) (http://www.animalgenome.org/QTLdb, Release 46, December 27, 2021), which includes 2605 QTL from 104 publications on 64 different traits. To detect the putative QTL under selection, we considered the QTLs that were located in genomic regions, including consecutive SNPs with a *q*-value lower than 0.05 or extended up to ± 40 kbps at both sides. We extracted the associated recorded genes for height in humans accessible on the genomewide association study (GWAS) catalog database (https://www.ebi.ac.uk/gwas) (Buniello et al. 2019) to identify new possible candidate genes for height variation in Iranian horse populations.

## Results

**Fig. 2:**
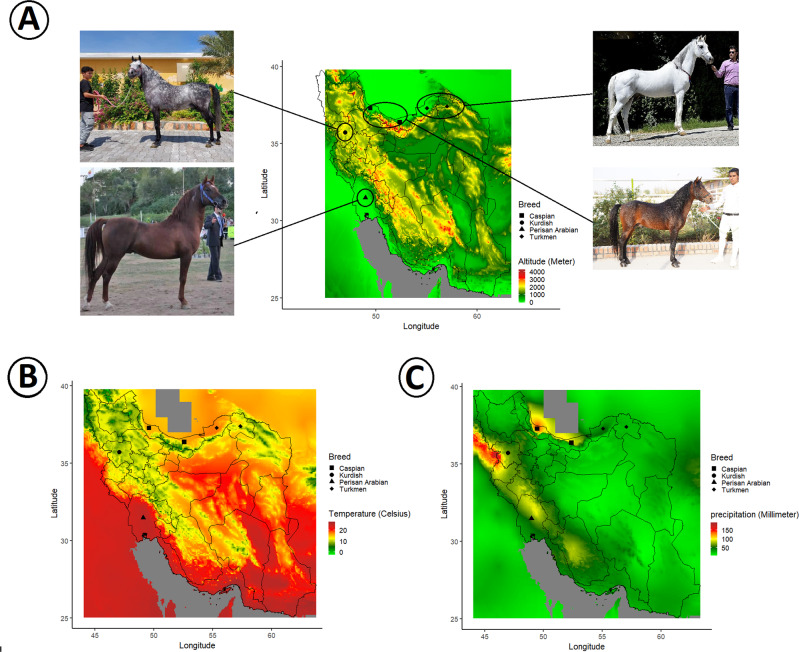
Iranian horse populations and their geographical locations. The geographical locations are shown based on altitude (**A**), average temperature (**B**), and average precipitation (**C**). The average temperature and precipitation were calculated based on the temperatures of 1970 to 2000 that were retrieved from the WorldClim version 2.1 climate database (https://www.worldclim.org/).

A total of 275 genotyped horse samples using two types of SNP assays from four data sets were included in this study (Supplemental file, Table S1). After QC, 4 and 83 samples were removed from the data due to the genotype rate and high relatedness, respectively. The four main Iranian horse breeds under study have their own characteristics (see Supplementary file, Table S2) and were developed in different environmental conditions (Fig. 2 and Supplementary file, Table S3). To quantify and control for the extreme deviation of a sample from samples of the same breeds, outliers were detected and removed based on the first three PCs (Supplementary file, Fig. S1). A total of 19 samples were excluded from the final data (Supplementary file, Fig. S2). More information about the outliers is available in Supplementary Table S4 and Fig. S3.

### Genetic distances, pairwise F_ST_, and AFD

Genetic distance analyses revealed that the Persian Arabian and Kurdish samples had the lowest value (Table 1). The Kurdish and Turkmen samples were intermediate in their genetic distance, followed by the Turkmen and Caspian samples. Persian Arabian horses showed the greatest divergence from the Caspian and Turkmen samples. Considering a 95% confidence interval, all breeds were distinguishable from one another with a pairwise F_ST_ >0 (Table 1). The Turkmen and Persian Arabian samples showed the highest divergence. The lowest pairwise F_ST_ observed between breeds originated from more geographically similar locations, including Turkmen vs. Caspian, and Kurdish vs. Persian Arabian. Considering AFD as a metric of genetic differentiation between the studied horse samples with asymmetrical sample size, the lowest magnitude of pairwise comparisons was in Kurdish vs. Persian Arabian (Table 1). The Kurdish showed similar AFD values to the Caspian and Turkmen horses, followed by the Turkmen and Caspian samples. Similar to the results for genetic distance, the highest AFD values were observed in the comparisons between Persian Arabian and the two Caspian and Turkmen breeds that were sampled in geographically distinct locations.

**Table 1:** Metrics of the Cavalli-Sforza and Edwards chord distance (upper triangle with absolute allele frequency difference ± SD within parentheses) and pairwise *F*_*ST*_ calculated (lower triangle with a 95% confidence interval within parentheses) from the SNP information.

	Caspian	Turkmen	Kurdish	Persian Arabian
Caspian	0	0.0170 (0.0877 ± 0.07056)	0.0174 (0.0854 ± 0.06904)	0.0228 (0.1122 ± 0.09163)
Turkmen	0.0039 (0.0037 to 0.004)	0	0.0159 (0.0858 ± 0.0685)	0.0216 (0.1111 ± 0.08908)
Kurdish	0.0044 (0.0043 to 0.0045)	0.0052 (0.0051 to 0.0053)	0	0.0123 (0.0727 ± 0.05954)
Persian Arabian	0.0099 (0.0097 to 0.0101)	0.0104 (0.0102 to 0.0106)	0.0039 (0.0039 to 0.004)	0

### Estimation of effective population size

**Fig. 3:**
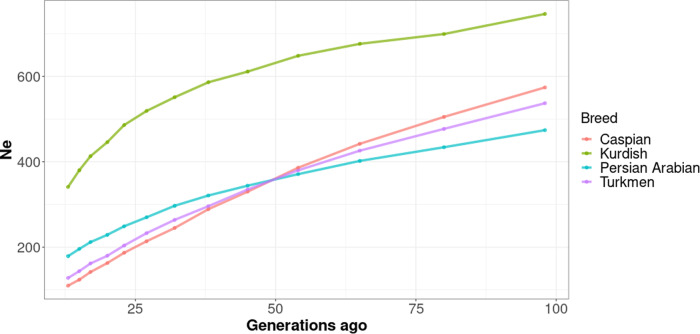
Estimates of historical effective population size (Ne). The estimates represent the Ne from 100 generations ago for each population analyzed based on the linkage disequilibrium (LD) method.

A decrease in *N*_*e*_ over time was apparent in all breeds, with a sharper decay for the more recent generations. The historical *N*_*e*_ pattern of the Caspian breed differed from that of the other studied breeds, with the Caspian breed having the lowest *N*_*e*_ in the recent generations (≤45 generations ago) and the second highest *N*_*e*_ in the older generations (≥54 generations ago) (Fig. 3). The Kurdish population showed the greatest *N*_*e*_.

On the basis of LD estimations, the contemporary *N*_*e*_ was estimated with various thresholds used to screen out rare alleles (Table 2). The lowest estimated contemporary *N*_*e*_ was for Turkmen with a range from 52.3 to 59.3. Values for the Caspian and Persian Arabian breeds, respectively, were similar: at 86.9 and 86 for MAF less than 0.1 and up to 98.4 and 101.7 using all SNPs. The Kurdish breed had the highest contemporary *N*_*e*_ compared to the other breeds.

**Table 2:** Estimated effective population size (jackknife confidence interval in parentheses) based on linkage disequilibrium (LD) information using different minor allele frequencies (MAFs) for alleles to be included in the analysis.

Breed	MAF < 0.1	MAF < 0.05	MAF < 0.02	All SNPs
Caspian	86.9 (47.8–317.5)	92.2 (50.4–357.1)	95.1 (52.2–361.4)	98.4 (54.2–372.0)
Turkmen	52.3 (30.7–117.2)	55.9 (32.9–126.1)	58.7 (35.0–129.3)	59.3 (35.5–129.3)
Kurdish	105.2 (76.1–156.5)	108.8 (78.9–161.8)	111.5 (80.9–165.8)	113.3 (82.4–167.8)
Persian Arabian	86.0 (60.9–133.6)	91.7 (65.7–140.4)	97.2 (70.0–147.8)	101.7 (73.7–153.1)

### Population genetic structure and linkage disequilibrium

**Fig. 4:**
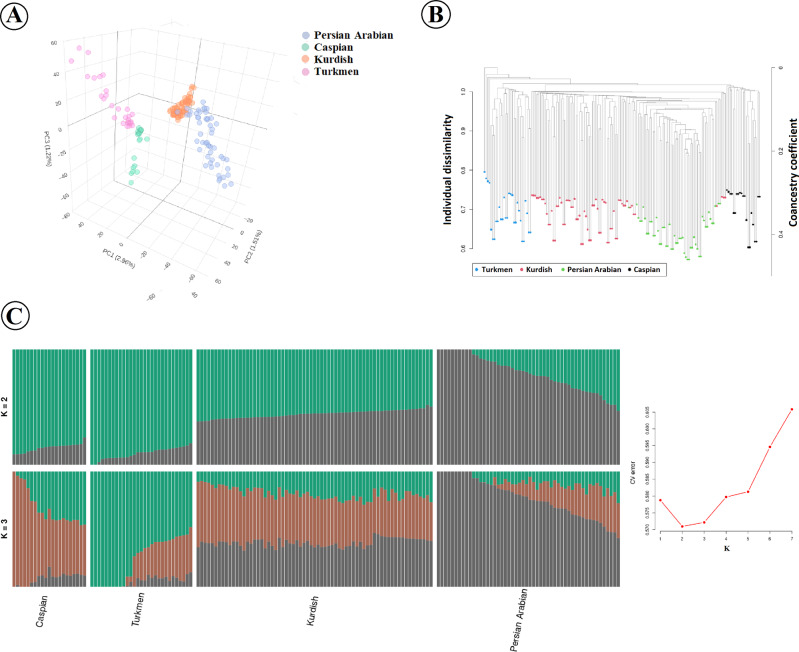
Population structure and relationship between Caspian, Turkmen, Persian Arabian, and Kurdish breeds tested in this study. **A** Principal component analysis based on the first three PCs. The four main Iranian breeds show genetic differentiation associated with their geographic origin clustered into two categories, including north of Iran (for Caspian and Turkmen) and southwest and/or west of Iran (Persian Arabian and Kurdish). **B** A relatedness tree was constructed using whole-genome SNP data. **C** Model-based clustering of four Iranian breeds using admixture analysis with the assumed number of ancestries of 2 and 3 (left) with a plot for CV errors of K ranging from 1 to 7 (right).

The first PC separated breeds were based on the two geographic origin clusters, including north of Iran (for Caspian and Turkmen) and southwest and/or west of Iran (Persian Arabian and Kurdish) (Fig. 4A). PC1 also helped distinguish between the Persian Arabian and Kurdish breeds. The second PC separated the breeds within the two geographic origins from each other. Furthermore, the Caspian breed was separated from the Turkmen breed by the third PC.

The relationships indicated in the relatedness analysis using identity-by-descent measures (Fig. 4B) were supported by the separation patterns that were identified using PCA. The Caspian and Turkmen branches were most proximal to each other compared to both the Persian Arabian and Kurdish breeds. Our analysis divided the Kurdish breed into two separate branches, suggesting distinct subpopulations within this breed. The presence of both Persian Arabian and Kurdish horses in several instances suggests either recent crossbreeding or shared ancestry among these horses.

Admixture analyses were performed by assuming up to 7 clusters (K) to identify the most likely number of ancestral populations as determined by the cross-validation (CV) algorithm. The results are shown in Fig. 4C (for K = 2 to 3) and Supplementary file, Fig. S5 (for K = 4 to 7). The lowest CV error was detected for the model with two ancestral populations, which was very close to the error detected for K = 3. In K = 2, the Caspian and Turkmen samples were assigned primarily to one genetic cluster and the Persian Arabian sample to the other; the Kurdish horses had a relatively equal assignment to both clusters. With K = 3, the Caspian and Turkmen samples became more distinct, but with evidence of population structure in the Turkmen sample, as some horses shared similar ancestry proportions to Caspian horses. The Persian Arabian horses remained primarily assigned to the third cluster, apart from the Caspian and Turkmen horses, but with some Persian Arabian horses having admixtures from the genetics largely represented by the Caspian horses. The Kurdish horses remained admixed, but horses of that breed showed similar proportions of genetic ancestry from each of the three genetic clusters. The admixture results for higher numbers of ancestral populations are shown in the Supporting file, Fig. S5.

The LD patterns between the studied breeds indicated that the mean of *r*^2^ in all breeds dropped rapidly up to approximately 100 kbps (Supplementary file, Fig. S4). The Persian Arabian population showed the slowest decay of LD compared to the other breeds. The average *r*^2^ at 100 kbps for all, Turkmen, Caspian, Kurdish, and Persian Arabian breeds were 0.143, 0.161, 0.151, 0.152, and 0.183, respectively.

### Runs of homozygosity (ROH)

A total of 60,408 ROH segments was identified in all the studied breeds (Supporting file, Table S5). As the sample size was different for the breeds, the absolute number of ROH in each breed is not comparable. The majority of ROH segments were classified in the shortest category with the length less than 8 Mb (> 97%). The total number of ROH segments in Kurdish, Persian Arabian, Turkmen, and Caspian horses were 24,194, 18,959, 9846, and 7409, respectively. The number of ROH segments exceeding 32 Mb length in Caspian, Kurdish, Persian Arabian, and Turkmen horses were 16, 13, 9, and 6 segments, respectively. Using a threshold of 0.7 for the shared ROH within each population, 24 peaks were found in Turkmen (2 peaks), Caspian (14 peaks), and Persian Arabian (8 peaks) populations (Supplementary file, Table S6).

Genomic inbreeding based on ROH (F_ROH_) was estimated for each population separately (Supplementary file, Fig. S6). The mean estimated genomic inbreeding was similar across breeds, ranging from 21% in the Turkmen population to 26% in the Persian Arabian population. The Kurdish and Caspian populations showed a very similar inbreeding with means of 22% and 23%, respectively.

### Selective signal and related GO and QTL detection

#### Caspian vs Turkmen

**Fig. 5:**
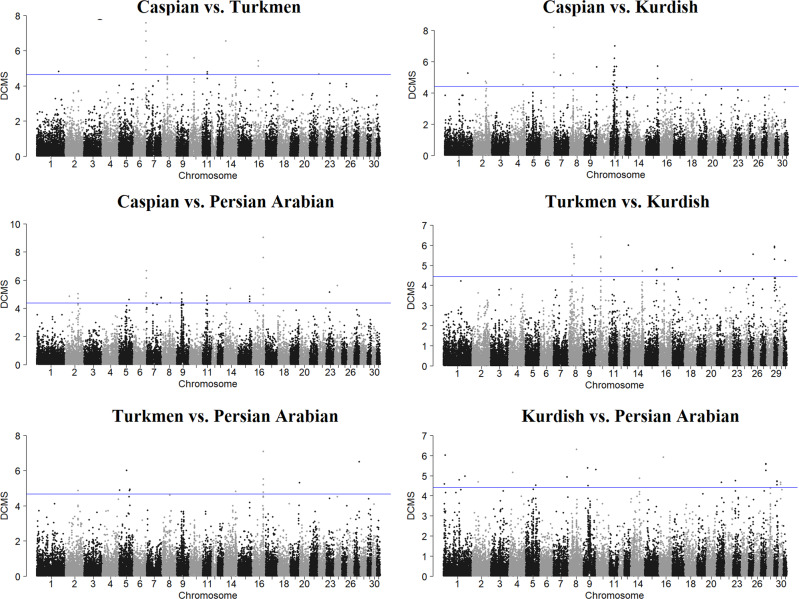
Manhattan plot of the -log10 (*P* values) calculated by the DCMS method in the six possible pairwise comparisons. The blue lines represent the significant threshold level at an FDR of 5% (i.e. *q*-value < 0.05).

We identified 13 significant SNPs located near to 12 genes (3 lncRNA, 1 miRNA, and 8 protein-coding genes) on 7 different chromosomes (chr) (chr1, chr6, chr8, chr10, chr11, chr14, and chr16) (Supplementary file, Table S7). The most significant SNP was identified on chr6 (82.8 Mb) (Fig. 5). Three of the nearby autosomal genes in *Equus caballus* (ECA) included *LLP homolog* (*LLPH*) (chr6: 82.8 mega base pairs (Mbps)), *high mobility group AT-hook 2* (*HMGA2*) (chr6: 82.5 Mbps), and *Musashi RNA Binding Protein 2* (*MSI2*) (chr11: 32.2 Mbps), previously shown to be associated with human height based on the GWAS catalog database (Supplementary file, Table S7). The results of gene enrichment analysis showed 17 significantly overrepresented GO terms in this comparison (Supplementary file, Table S9). The most significant term was GO:0004950 (chemokine receptor activity). Some of the previously identified QTLs were found in the suggestive regions of selection in this comparison (Supplementary file, Table S7). We found 3 significant SNPs close to 6 different QTLs classified into 4 types, including wither height (*n* = 2), alternate gaits (*n* = 1), chronic progressive lymphedema (*n* = 2), and osteochondrosis QTL (*n* = 1).

#### Caspian vs. Kurdish

A total of 27 significant SNPs were identified in this comparison (Supplementary file, Table S7). The most significant SNPs were located close to *LLPH* (chr6: 82.8 Mbps) with a DCMS value of 14.86 (Fig. 5). Gene annotation analysis of the significant SNPs in this pairwise comparison detected 45 (6 lncRNA, 2 miRNA, 1 pseudogene, and 36 protein-coding genes) candidate genes. Based on the GWAS catalog database, 19 of the candidate genes have been associated with human height. We also found 6 significant SNPs with at least one QTL located near to them in 5 different QTL types, including insect bite hypersensitivity (*n* = 2), wither height (*n* = 4), hair density (*n* = 1), alternate gaits (*n* = 1), and navicular bone morphology (*n* = 1). Of those, two suggested QTLs under selection for wither height located on chr11 (with QTL IDs 166105 and 165767) did not show selection pressure in the two other Caspian pairwise comparisons. No GO terms were significantly overrepresented in this comparison.

#### Caspian vs. Persian Arabian

We identified 24 significant SNPs in this comparison, with the most significant SNP on chr16 (71.8 Mbps) with a DCMS value of 14.59. Gene annotation analysis of the significant SNPs detected 28 (8 lncRNA, 1 miRNA, 1 snRNA, and 18 protein-coding genes) candidate genes. Of them, 9 candidate genes were found in the GWAS catalog list. The results showed 19 significantly overrepresented GO terms for the Caspian vs. Persian Arabian comparison. The most significant GO term was GO:0016790 (thiolester hydrolase activity). Among the significant SNPs, 4 loci were located close to 7 QTLs, which were classified into 5 QTL types, including immunoglobulin E level (*n* = 1), wither height (*n* = 2), alternate gaits (*n* = 2), equine sarcoids (*n* = 1), and immunoglobulin G level (*n* = 1).

#### Turkmen vs. Kurdish

Based on the DCMS method, 25 significant SNPs were detected on 10 different chromosomes (chr8, chr10, chr13, chr14, chr15, chr17, chr21, chr25, chr29, and chr31), with the most significant SNPs on chr10 (32.7 Mbps). Gene annotation analysis of these significant SNPs resulted in 28 candidate genes (4 lncRNA, 1 snoRNA, 1 snRNA and 22 protein-coding genes). Of those, 11 candidate genes were located in the GWAS catalog database for human height. Gene enrichment analysis of the candidate genes detected 2 significantly overrepresented terms that were classified as belonging to the cellular component domain. The comparison detected only 1 significant SNP close to 2 QTLs that was classified as type 1, navicular bone morphology.

#### Turkmen vs. Persian Arabian

We identified a total of 13 significant SNPs on chr2, chr5, chr14, chr16, chr19, and chr27. The most significant SNP based on the DCMS method was located on chr27 (37.0 Mbps). Gene annotation analysis of these significant SNPs found 15 candidate genes for the pairwise comparison. Of them, 7 genes were found in the GWAS catalog list for human height. Gene enrichment analysis of the candidate genes detected 29 significantly overrepresented terms in the comparison, while the four top significant terms were related to the molecular function domain. In the comparison, 2 of 13 significant loci were located close to 3 QTLs, which were classified into 3 types, including immunoglobulin E level (*n* = 1), osteochondritis dissecans (*n* = 1), and osteochondrosis (*n* = 1).

#### Kurdish vs. Persian Arabian

Here, 29 SNPs with *q*-values less than 0.05 were detected, with the most significant signal on chr8 (52.2 Mbps). A total of 31 candidate genes was detected in the gene annotation analysis in the comparison. Of them, 9 genes were located in the GWAS catalog list. One of these, *TGFB2* (chr30: 143 Mbps), has been noted as a candidate for human height in 10 different GWAS studies (Akiyama et al. 2019). *TGFB* is a growth factor and key regulator of several traits related to body composition, growth, and development in chicken (Dadousis et al. 2021; Li et al. 2003). The second candidate gene was *trafficking protein particle complex 9* (*TRAPPC9*) (chr9: 81.6 Mbps). The other 7 genes were *ABRAXAS2* (chr1: 8.3 Mbps), *GABPB1* (chr1: 140.9), *NREP* (chr14: 59.7 Mbps), *STARD4* (chr14: 59.8 Mbps), *RORB* (chr23: 17.1 Mbps), *Cub and Sushi Multiple Domains 1* (*CSMD1*) (chr27: 37.0 Mbps), and *CAMK1D* (chr29: 23.1 Mbps), which have also been associated with human height. Gene enrichment analysis of candidate genes from Kurdish vs. Persian Arabian comparison found two significantly overrepresented terms in the molecular function domain (FDR-adjusted *P*-value < 0.05). The results showed that 7 of 28 significant loci were located close to at least one QTL. These nearby QTLs had one of three classifications, including Guttural pouch tympany (*n* = 1, chr2: 39.4 Mbps), wither height (*n* = 3, chr8: 52.2 Mbps and chr9: 28.6 Mbps), and navicular bone morphology (*n* = 1, chr29: 23.0 and 23.2 Mbps).

### Observed similarities between pairwise comparisons

#### SNPs

We found 10 significant SNPs that were common in two comparisons, including Caspian vs. Kurdish and Caspian vs. Persian Arabian. Of these 10, 4 SNPs were also significant in the Turkmen vs. Caspian comparison, located on chr6 from 82.4 to 82.8 Mbps (Supplementary file, Table S7). Only 1 SNP located on chr14 was significant in both Turkmen vs. Persian Arabian and Turkmen vs. Kurdish comparisons. Our results showed 3 common significant SNPs in Turkmen vs. Caspian and Turkmen vs. Kurdish comparisons located on chr8 (38.0 and 38.1 Mbps) and chr10 (31.2 Mbps). None of the significant SNPs overlapped in the three comparisons of Turkmen vs all others. We found 2 significant SNPs in Kurdish vs. Caspian and Kurdish vs. Turkmen comparisons located on chr8 (25.9 Mbps) and chr15 (76.6 Mbps). The results showed 3 significant SNPs on chr16 that were common in the comparisons of the Persian Arabian with both the Caspian and Turkmen populations. There were 3 common SNPs with *q*-values less than 0.05 on chr5 (*n* = 1) and chr27 (*n* = 2) in both Persian Arabian vs. Kurdish and Persian Arabian vs. Turkmen comparisons.

#### Genes

**Fig. 6:**
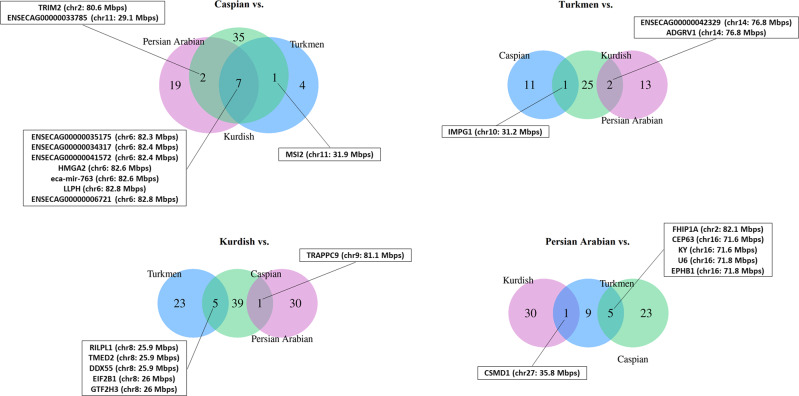
Venn diagrams of overlapped candidate genes. The plot summarizes the number of overlapped candidate genes under selection among different pairwise comparisons.

We identified 8, 7, and 9 candidate genes overlapping in Caspian pairwise comparisons for Turkmen and Kurdish, Turkmen and Persian Arabian, and Kurdish and Persian Arabian analyses, respectively (Fig. 6). The results showed that 7 candidate genes were present in all the three Caspian pairwise comparisons. One of the significant SNPs, MNEc.2.6.81451782.BIEC2-1024200, was located within *HMGA2*. The allele G was the most common in the Caspian population with 0.95 frequency, while the frequencies for Turkmen, Kurdish, and Arabian populations were 0.33, 0.35, and 0.23, respectively (Supplementary file, Table S8). Our gene annotation analysis for the significant SNPs in all the three Caspian comparisons also detected another overlapped gene, *LLPH*, which was a candidate for selection in the Caspian vs. Kurdish comparison. We also identified a candidate gene located in the GWAS catalog list for human height, *TRIM2* (chr2: 80.8 Mbps), which was common in the pairwise comparisons between Caspian and both Kurdish and Persian Arabian populations. There were 6 and 14 additional candidate genes for the pairwise comparisons between Caspian and both Persian Arabian and Kurdish populations, respectively, which were located in the GWAS catalog list for human height (Supplementary file, Table S7).

We found 1 gene, *IMPG1* (chr10: 31.2 Mbps), overrepresented in the comparisons between Turkmen and both Caspian and Kurdish populations, which is an ortholog for a protein-coding gene in humans and is a major component of the *interphotoreceptor matrix* (*IPM*). The IPM is suggested to have many important functions related to growth factors, such as regulation of retinoid transport, participation in cytoskeletal organization in the surrounding cells, and regulation of oxygen and nutrient transport (Ishikawa et al. 2015). Investigation of the genes for selective pressure in the comparisons between Turkmen and both Persian Arabian and Kurdish populations showed two common genes, ENSECAG00000042329 and *U6* (chr29: 6.9 Mbps). *U6* was associated with human height in 16 different GWASs (Kichaev et al. 2019; Sakaue et al. 2021).

There were 5 common genes, including ENSECAG00000006191 (chr8: 25.9 Mbps), *transmembrane emp24 protein transport domain containing 2* (*TMED2*) (chr8: 25.9 Mbps), *DDX55* (chr8: 25.9 Mbps), *eukaryotic initiation factor 2B 1* (*EIF2B1*) (chr8: 25.9 Mbps), and *GTF2H3* (chr8: 25.9 Mbps), in the comparisons between Kurdish and both Turkmen and Caspian breeds. We also detected one gene, *trafficking protein particle complex 9* (*TRAPPC9*) (chr9: 81.6 Mbps), that was common in the comparisons between Kurdish and both Persian Arabian and Caspian breeds.

The common SNPs resulted in identification of 5 nearby genes in the comparisons between Persian Arabian and both Turkmen and Caspian breeds. There was also 1 common candidate gene, *CSMD1* (chr27: 35.8 Mbps), listed in the GWAS catalog database, in the comparisons between Persian Arabian and both Turkmen and Kurdish breeds. *CSMD1* is a large transmembrane complement inhibitor with expression in many tissues such as testis, brain, lung, colon, thyroid gland, breast, and pancreas (Fagerberg et al. 2014). This gene is associated with several pathological processes, from neurodegenerative and psychiatric disorders to infertility and cancer (Gialeli et al. 2021). The comparisons between Persian Arabian and both Caspian and Turkmen breeds showed another 4 overrepresented candidate genes, *CEP63* (chr16: 71.5 Mbps), *kyphoscoliosis peptidase* (*Ky*) (chr16: 71.8 Mbps), *EPHB1* (chr16: 71.8 Mbps), and *U6*, which were listed in the GWAS catalog for human height.

#### QTLs

Some of the previously identified QTLs were found in the suggestive regions of selection in each of the comparisons (Supplementary file, Table S7). Among the different QTL types found in the Caspian pairwise comparisons, 2 QTLs (with QTL IDs 28316 and 28297) related to wither height and one QTL (with QTL ID 165888) related to alternate gaits were common in the comparisons between Caspian and all the other breeds (Supplementary file, Fig. S7). Concerning the QTL types in the three comparisons for Turkmen, the QTL for osteochondrosis was common in Turkmen vs. Caspian (with QTL ID 27159) and Turkmen vs. Persian Arabian (with QTL ID 28236) comparisons. Navicular bone morphology QTL type was common in all the three comparisons between Kurdish and three other breeds. Immunoglobulin E level QTL type (with QTL ID 28467) was represented in both Persian Arabian vs. Turkmen and Persian Arabian vs. Caspian comparisons. In addition, wither height QTLs were detected in comparisons between the Persian Arabian and both the Caspian and Kurdish samples.

#### GO

The results showed 17 and 19 significantly overrepresented GO terms for Caspian vs. Turkmen and Caspian vs. Persian Arabian comparisons, respectively, while the only GO:0060999 term (positive regulation of dendritic spine development) was present in these two comparisons (Supplementary file, Fig. S8). One GO term, GO:0015026 (coreceptor activity), was common in two Turkmen vs. Persian Arabian and Turkmen vs. Caspian comparisons. There were no significantly overrepresented GO terms in the three comparisons between Kurdish and the other breeds. A total of 10 GO terms were overrepresented in both Persian Arabian vs. Turkmen and Persian Arabian vs. Caspian comparisons. Two of these common GO terms, GO:0060996 (dendritic spine development) and GO:0060997 (dendritic spine morphogenesis), are related to the dendritic spine.

## Discussion

A better understanding of the effects of the evolution and adaptability of livestock species to different environments can improve our knowledge about the selection process (Saravanan et al. 2022). Iranian horse populations have been subject to different breeding objectives in distinct geographic regions with variation in the components of the environment such as temperature, humidity, and altitude (Fig. 2 and Supplementary file, Table S3). Our findings showed a genetic division among the studied breeds that matched their different geographic origins. These results refine our understanding of the genetic relationship between these four Iranian horse breeds. We also identified genomic regions under selective pressures, and that these regions previously had been reported to be associated with morphology, adaptation, and fitness (Frischknecht et al. 2015; Smith et al. 2007), which are important phenotypic traits in horse breeding. Therefore, these results can support future genomewide association studies and subsequent fine mapping of important traits to improve our understanding of the genetic basis of these traits and allow for improved horse breeding.

### Effective population size

Simplified, *N*_*e*_ corresponds to the number of breeding animals in a population under idealized conditions (Wright 1931). Reduction in *N*_*e*_, as a result of decrease of genetic diversity within a population, may significantly reduce the population’s ability to adapt to perturbations such as extreme weather conditions, disease, and infections (Palstra and Ruzzante 2008), and this may in turn increase the risk of extinction (Newman and Pilson 1997). The contemporary *N*_*e*_ estimations performed within this study indicate that all four horse breeds should be considered for conservation, most urgently for the Turkmen horse breed, which showed the lowest *N*_*e*_ with a value close to 50. The historical *N*_*e*_ values computed were of the same magnitude as those reported in other studies using similar approaches (Corbin et al. 2010; Jasielczuk et al. 2020; Nazari et al. 2022; Salek Ardestani et al. 2022). Our study represents the first estimates of contemporary *N*_*e*_ for the Turkmen, Caspian, Kurdish, and Persian Arabian populations, and alongside, the other studies that estimated their historical *N*_*e*_ can help to quantify the magnitude of genetic drift and inbreeding in real-world Iranian horse populations (Nazari et al. 2022; Salek Ardestani et al. 2022).

### Population genetic structure

A clear genetic division among 36 globally distributed horse breeds was previously detected using a genomewide set of autosomal SNPs, and this genetic diversity largely reflected geographic origins and known breed histories (Petersen et al. 2013). Similarly, our results well matched the studied breeds with different geographic origins, as we classified the north breeds (Caspian and Turkmen) and southwest and west breeds (Persian Arabian and Kurdish) into two phylogeographic clades. These results are consistent with the results from Sadeghi et al. (2019), in which the PCA results demonstrated that the genetic variation was associated with the separation among indigenous horse breeds from different parts of Iran. We observed close genetic relationships between the Iranian horse breeds originating from the same geographic region. In concordance, relatedness and admixture analysis showed a close genetic relationship between Persian Arabian and Kurdish breeds, as well as between Turkmen and Caspian breeds.

### Inbreeding

VanRaden et al. (2011) showed that pedigree measures of inbreeding values are not equivalent to the genomic inbreeding values. This difference may lead to an imperfect reflection of the level of homozygosity in the genome. ROH, the indicator of genomic autozygosity, are used more often to estimate the degree of realized genomic inbreeding (Chhotaray et al. 2021; Dadousis et al. 2022; Eydivandi et al. 2021b; Metzger et al. 2015; Saravanan et al. 2021). On assessing different genomewide SNP-based estimators of inbreeding using computer simulations, F_ROH_ provided a more precise estimate of inbreeding than could be expected by using pedigrees (Caballero et al. 2022). On comparing different marker-based estimators, F_ROH_ showed the highest correlation with pedigree-based coefficients in cattle using imputed SNP data (Dadousis et al. 2022). Long homozygous stretches and consequently high inbreeding coefficients characterize closed populations or those derived from a relatively narrow genetic base such as the Arabian and Thoroughbred populations (Metzger et al. 2015). In concordance, the Persian Arabian horses in our study showed a relatively high number of larger ROH (with sizes > 4 Mb, see Supplementary file, Table S5), probably due to the more recent inbreeding, as the ROH will break up over time by recombination, and consequently a relatively higher value for F_ROH_, indicative of inbreeding. Thus, when considering matings at an individual animal level, genomic information can provide a more accurate measure of inbreeding (homozygosity across the genome). This information can be applied in mating systems with the aim of minimizing inbreeding depression, especially in the Persian Arabian horses, which showed the highest estimated genomic inbreeding value compared with the other Iranian breeds.

### Genomic signatures of local adaptation

The genomes of local breeds have been under natural and artificial selection for centuries to transmit the desired properties to the descendants. These genetic changes shaped recent gene pools, and can be used for investigating the genetic relatedness among breeds (Eydivandi et al. 2021a; Ju et al. 2019; Rajawat et al. 2022). Our study showed that the genomes of Iranian horse breeds contain multiple genomic regions under selective pressures (adaptation and/or breeding), representing an opportunity for a more detailed investigation of the genes involved in these procedures. For instance, *ADGRV1* (chr14: 76.8) was under selective pressure in the Arabian and Kurdish comparison. This gene is suggested to have highly differentiated nonsynonymous alleles, which leads to an excess of the adrenocorticotropic hormone in Yakutian horses, which are known to be highly adapted to subarctic environments (Librado et al. 2015). Another candidate gene, *TMED2*, has been reported to regulate innate immune signaling and to specialize in responding to environmental exposures (Sun et al. 2018). Additionally, *EIF2B1* is associated with stress signaling, as inhibition of this gene mediates the downregulation of protein synthesis under stress (Slynko et al. 2021). *TRAPPC9* was common in comparisons between Kurdish and both Persian Arabian and Caspian breeds. This has been reported to be important in the trafficking and signaling pathways in health and disease in humans, where mutations in *TRAPPC9* are linked to a form of mental retardation, breast and colon cancer, and liver diseases (Mbimba et al. 2018). Among the 4 common candidate genes from comparisons between Persian Arabian and both Caspian and Turkmen breeds, *Ky* is a gene involved in muscle growth, as the absence of *Ky* protein leads to muscular dystrophy in mouse (Blanco et al. 2001). The suggested selective sweep regions were close to or overlapped immunoglobulins, insect bite hypersensitivity, and guttural pouch tympany, while other sweeps overlapped wither height and alternate gaits. This suggests that sweep regions may help to understand the biology of these breeds and to improve our understanding of the changes in the biological domain as a result of variation in the identified genes, some of which were related to an important QTL. Moreover, this information can be used to develop breeds or crossbreeds that have a better performance and a higher environmental tolerance based on the detected candidate genes.

### Genomic signatures of height

The Caspian horse is thought to be an ancient breed native to the north of Iran. This horse is considered as a small horse with height ranging from 9 to 10 hands (Shahsavarani and Rahimi-Mianji 2010). In accordance with these studies, our selection signatures revealed the *HMGA2* in a genomic region putatively under selective pressure in all comparisons between Caspian as a small horse and the others with standard height. It has been shown that *HMGA2* has a crucial role in the size variation of horses. For instance, investigation of the wither height in Shetland ponies by genomewide association (GWAS) using the GGP equine SNP70 BeadChip detected *HMGA2* as a major QTL for this trait (Frischknecht et al. 2015). The study showed that the height of Shetland ponies and other small horses was reduced due to a nonsynonymous mutation in *HMGA2*. This gene has a well-known role in height determination in other species, including humans (Buysse et al. 2009) and dogs (Rimbault et al. 2013). The GWAS catalog data included 22 different studies that showed association between *HMGA2* and the human height trait (Supplementary file, Table S7). This gene also showed a pleiotropic effect on both height and metabolic traits in ponies (Norton et al. 2019). Our results showed that the selection signal for this gene was detected due to a high frequency for allele G in the intragenic SNP MNEc.2.6.81451782.BIEC2-1024200 compared to the other studied breeds. Another gene in the same region overrepresented in all the three Caspian pairwise comparisons was *LLPH*, previously associated with height in human studies based on the GWAS catalog database. A GWAS in Duroc pigs showed a significant association between the average daily gain and a haplotype block containing *LLPH* (Quan et al. 2018). Although the four loci can explain up to 83% of the height variance in horses (Makvandi-Nejad et al. 2012), most of the underlying genetic variants that affect the height remain unknown. To pave the way for understanding the molecular mechanisms of height in Iranian horse populations, we suggest the regions that contain *HMGA2* and *LLPH* as strong candidates for the height variation between Caspian and the other Iranian breeds. Beyond these genes, we suggest 38 new putative candidate genes under selective pressure that are related to variation in height traits in humans.

### QTLs

Each Iranian horse breed has its own historical background and differs in usage. Finding selection signatures close to QTLs indicates a relationship between the selection for traits and the effects of variation at a locus. Accordingly, we investigated the candidate QTLs and biological pathways within the candidate regions under selection to identify pathways that might have shaped the Iranian horse genome. Some of the SNPs identified under selection were located close to previously reported QTLs related to morphological and behavioral traits in horses, such as wither height, hair density, navicular bone morphology, and alternate gaits. Some of them were located near to QTLs related to adaptation traits such as immunoglobulin level (Smith et al. 2007) (i.e. E and G) and insect bite hypersensitivity (Ablondi et al. 2020). Most of these SNPs were located near QTLs related to genetic disorders such as guttural pouch tympany, osteochondritis dissecans, osteochondrosis, and chronic progressive lymphedema. These results highlight that the suggested genes and QTLs within these categories might be targeted by selection in Iranian horses to adapt to environmental conditions or respond to human-mediated selection.

## Conclusions

In general, exploring the genetic variation between Iranian horse populations evolved under diverse ecological conditions with prominent phenotypic variation suggested a clear genetic division among the breeds, which largely reflects their geographic origins. The use of information obtained from extensive studies in humans, retrieved from the GWAS catalog, can help to better interpret the results of selection signature studies. This information helped us to introduce 38 new candidate genes for height variation in Iranian horse populations in addition to the well-known regions that contain *HMGA2* and *LLPH* as strong candidates. In addition, the identified candidate sweep regions provide a genomewide map of selection signatures in the studied breeds, including genes and QTLs for known morphological, adaptation, and fitness traits. This information may be valuable in formulating genetic conservation and improved breeding strategies for these populations.

## Supplementary information

Supplementary file

## Data Availability

The Sadeghi et al. 2019 and Cosgrove et al. 2020 data analyzed during the current study are publicly available at dryad (https://datadryad.org/stash/dataset/doi:10.5061/dryad.54vb7f2) and Mendeley Data (https://data.mendeley.com/datasets/mkk5khxrbp/3), respectively. All merged genotypic data used in this study are available at dryad (10.5061/dryad.37pvmcvqr).

## References

[CR1] Ablondi M, Dadousis C, Vasini M, Eriksson S, Mikko S, Sabbioni A (2020). Genetic diversity and signatures of selection in a native Italian horse breed based on SNP data. Animals.

[CR2] Akiyama M, Ishigaki K, Sakaue S, Momozawa Y, Horikoshi M, Hirata M (2019). Characterizing rare and low-frequency height-associated variants in the Japanese population. Nat Commun.

[CR3] Ala-Amjadi M, Yeganeh H, Sadeghi M (2017). Study of Genetic variation in Iranian Kurdish horse using microsatellite marker. Iran J Anim Sci.

[CR4] Alexander DH, Lange K (2011). Enhancements to the ADMIXTURE algorithm for individual ancestry estimation. BMC Bioinforma.

[CR5] Alexander DH, Novembre J, Lange K (2009). Fast model-based estimation of ancestry in unrelated individuals. Genome Res.

[CR6] Amjadi MA, Yeganeh HM, Sadeghi M, Raza SHA, Yang J, Najafabadi HA (2021). Microsatellite analysis of genetic diversity and population structure of the Iranian Kurdish Horse. J Equine Vet Sci.

[CR7] Barbato M, Orozco-terWengel P, Tapio M, Bruford MW (2015). SNeP: a tool to estimate trends in recent effective population size trajectories using genome-wide SNP data. Front Genet.

[CR8] Benjamini Y, Hochberg Y (1995). Controlling the false discovery rate: a practical and powerful approach to multiple testing. J R Stat Soc: Ser B (Methodol).

[CR9] Berner D (2019). Allele frequency difference AFD^−^An intuitive alternative to F(ST) for quantifying genetic population differentiation. Genes (Basel).

[CR10] Biscarini F, Cozzi P, Gaspa G, Marras G (2018). detectRUNS: Detect Runs of homozygosity and runs of heterozygosity in diploid genomes. R package version 0.9.5. 2018. Retrieved from https://CRAN.R-project.org/package=detectRUNS

[CR11] Blanco G, Coulton GR, Biggin A, Grainge C, Moss J, Barrett M (2001). The kyphoscoliosis (ky) mouse is deficient in hypertrophic responses and is caused by a mutation in a novel muscle-specific protein. Hum Mol Genet.

[CR12] Bonhomme M, Chevalet C, Servin B, Boitard S, Abdallah J, Blott S (2010). Detecting selection in population trees: the Lewontin and Krakauer test extended. Genetics.

[CR13] Breunig MM, Kriegel H-P, Ng RT, Sander J (2000). LOF: identifying density-based local outliers. In *Proceedings of the 2000 ACM SIGMOD international conference on management of data***.** 93-104.

[CR14] Browning SR, Browning BL (2007). Rapid and accurate haplotype phasing and missing-data inference for whole-genome association studies by use of localized haplotype clustering. Am J Hum Genet.

[CR15] Buniello A, MacArthur JAL, Cerezo M, Harris LW, Hayhurst J, Malangone C (2019). The NHGRI-EBI GWAS catalog of published genome-wide association studies, targeted arrays and summary statistics 2019. Nucleic Acids Res.

[CR16] Buysse K, Reardon W, Mehta L, Costa T, Fagerstrom C, Kingsbury DJ (2009). The 12q14 microdeletion syndrome: additional patients and further evidence that HMGA2 is an important genetic determinant for human height. Eur J Med Genet.

[CR17] Caballero A, Fernández A, Villanueva B, Toro MA (2022). A comparison of marker-based estimators of inbreeding and inbreeding depression. Genet Sel Evol.

[CR18] Chhotaray S, Panigrahi M, Pal D, Ahmad SF, Bhanuprakash V, Kumar H (2021). Genome-wide estimation of inbreeding coefficient, effective population size and haplotype blocks in Vrindavani crossbred cattle strain of India. Biol Rhythm Res.

[CR19] Corbin LJ, Blott SC, Swinburne JE, Vaudin M, Bishop SC, Woolliams JA (2010). Linkage disequilibrium and historical effective population size in the Thoroughbred horse. Anim Genet.

[CR20] Cosgrove EJ, Sadeghi R, Schlamp F, Holl HM, Moradi-Shahrbabak M, Miraei-Ashtiani SR (2020). Genome diversity and the origin of the Arabian Horse. Sci Rep..

[CR21] Dadousis C, Ablondi M, Cipolat-Gotet C, van Kaam J-T, Marusi M, Cassandro M (2022). Genomic inbreeding coefficients using imputed genotypes: Assessing different estimators in Holstein-Friesian dairy cows. J Dairy Sci.

[CR22] Dadousis C, Somavilla A, Ilska JJ, Johnsson M, Batista L, Mellanby RJ (2021). A genome-wide association analysis for body weight at 35 days measured on 137,343 broiler chickens. Genet Sel Evol.

[CR23] Danecek P, Auton A, Abecasis G, Albers CA, Banks E, DePristo MA (2011). The variant call format and VCFtools. Bioinformatics.

[CR24] Do C, Waples RS, Peel D, Macbeth GM, Tillett BJ, Ovenden JR (2014). NeEstimator v2: re-implementation of software for the estimation of contemporary effective population size (Ne) from genetic data. Mol Ecol Resour.

[CR25] Eydivandi S, Roudbar MA, Ardestani SS, Momen M, Sahana G (2021). A selection signatures study among Middle Eastern and European sheep breeds. J Animal Breed Gen.

[CR26] Eydivandi S, Roudbar MA, Karimi MO, Sahana G (2021). Genomic scans for selective sweeps through haplotype homozygosity and allelic fixation in 14 indigenous sheep breeds from Middle East and South Asia. Sci Rep.

[CR27] Fagerberg L, Hallström BM, Oksvold P, Kampf C, Djureinovic D, Odeberg J (2014). Analysis of the human tissue-specific expression by genome-wide integration of transcriptomics and antibody-based proteomics. Mol Cell Proteom.

[CR28] Fariello MI, Boitard S, Naya H, SanCristobal M, Servin B (2013). Detecting signatures of selection through haplotype differentiation among hierarchically structured populations. Genetics.

[CR29] Firouz L (1998). The original ancestors of the Turkoman, Caspian horses. *First international conference on Turkoman horses. Ashgabat, Turkmenistan*.

[CR30] Forbis J (1976). *The classic Arabian horse*. Liveright Publishing Corporation: New York.

[CR31] Fotovati A (2000). Persian horse breeds from ancient time to present and their rules in development of world horse breeds. ASIAN-AUSTRALASIAN JOURNAL ANIMAL SCIENCES.

[CR32] Frischknecht M, Jagannathan V, Plattet P, Neuditschko M, Signer-Hasler H, Bachmann I (2015). A non-synonymous HMGA2 variant decreases height in shetland ponies and other small horses. PLOS ONE.

[CR33] Gautier M, Vitalis R (2012). rehh: an R package to detect footprints of selection in genome-wide SNP data from haplotype structure. Bioinformatics.

[CR34] Gharahveysi S, Irani M (2011). Inbreeding study on the Iranian Arab horse population. World J Zool.

[CR35] Gialeli C, Tuysuz EC, Staaf J, Guleed S, Paciorek V, Mörgelin M (2021). Complement inhibitor CSMD1 modulates epidermal growth factor receptor oncogenic signaling and sensitizes breast cancer cells to chemotherapy. J Exp Clin Cancer Res.

[CR36] Goudet J (2005). Hierfstat, a package for R to compute and test hierarchical F‐statistics. Mol Ecol notes.

[CR37] Hu Z-L, Fritz ER, Reecy JM (2007). AnimalQTLdb: a livestock QTL database tool set for positional QTL information mining and beyond. Nucleic Acids Res.

[CR38] Ishikawa M, Sawada Y, Yoshitomi T (2015). Structure and function of the interphotoreceptor matrix surrounding retinal photoreceptor cells. Exp Eye Res.

[CR39] Jasielczuk I, Gurgul A, Szmatoła T, Semik-Gurgul E, Pawlina-Tyszko K, Stefaniuk-Szmukier M (2020). Linkage disequilibrium, haplotype blocks and historical effective population size in Arabian horses and selected Polish native horse breeds. Livest Sci.

[CR40] Jiskrová I, Vrtková I, Prausová M (2016). Genetic diversity of populations of Akhal-Teke horses from the CzechRepublic, Russia, Estonia and Switzerland. Acta Universitatis Agriculturae et Silviculturae Mendelianae Brunensis.

[CR41] Ju M-M, Feng L, Yang J, Yang Y-C, Chen X-D, Zhao G-F (2019). Evaluating Population Genetic Structure and Demographic History of Quercus spinosa (Fagaceae) Based on Specific Length Amplified Fragment Sequencing. Frontiers in Genetics.

[CR42] Kalbfleisch TS, Rice ES, DePriest MS, Walenz BP, Hestand MS, Vermeesch JR (2018). Improved reference genome for the domestic horse increases assembly contiguity and composition. Commun Biol.

[CR43] Kasprzyk A (2011). BioMart: driving a paradigm change in biological data management. Database.

[CR44] Kichaev G, Bhatia G, Loh P-R, Gazal S, Burch K, Freund MK (2019). Leveraging Polygenic Functional Enrichment to Improve GWAS Power. Am J Hum Genet.

[CR45] Lewontin RC, Krakauer J (1973). Distribution of gene frequency as a test of the theory of the selective neutrality of polymorphisms. Genetics.

[CR46] Li H, Deeb N, Zhou H, Mitchell AD, Ashwell CM, Lamont SJ (2003). Chicken quantitative trait loci for growth and body composition associated with transforming growth factor-beta genes. Poult Sci.

[CR47] Librado P, Der Sarkissian C, Ermini L, Schubert M (2015). Tracking the origins of Yakutian horses and the genetic basis for their fast adaptation to subarctic environments. **112**(50): E6889-E6897. 10.1073/pnas.1513696112 PMC4687531 26598656

[CR48] Lotterhos KE, Card DC, Schaal SM, Wang L, Collins C, Verity B (2017). Composite measures of selection can improve the signal-to-noise ratio in genome scans. Methods Ecol Evolution.

[CR49] Lotterhos KE, Card DC, Schaal SM, Wang L, Collins C, Verity B (2017). Composite measures of selection can improve the signal‐to‐noise ratio in genome scans. Methods Ecol Evolution.

[CR50] Lotterhos KE, Whitlock MC (2015). The relative power of genome scans to detect local adaptation depends on sampling design and statistical method. Mol Ecol.

[CR51] Ma Y, Ding X, Qanbari S, Weigend S, Zhang Q, Simianer H (2015). Properties of different selection signature statistics and a new strategy for combining them. Heredity.

[CR52] Makvandi-Nejad S, Hoffman GE, Allen JJ, Chu E, Gu E, Chandler AM (2012). Four loci explain 83% of size variation in the horse. PLoS One.

[CR53] Malomane DK, Reimer C, Weigend S, Weigend A, Sharifi AR, Simianer H (2018). Efficiency of different strategies to mitigate ascertainment bias when using SNP panels in diversity studies. BMC Genomics.

[CR54] Mbimba T, Hussein NJ, Najeed A, Safadi FF (2018). TRAPPC9: Novel insights into its trafficking and signaling pathways in health and disease. Int J Mol Med.

[CR55] Metzger J, Karwath M, Tonda R, Beltran S, Águeda L, Gut M (2015). Runs of homozygosity reveal signatures of positive selection for reproduction traits in breed and non-breed horses. BMC Genomics.

[CR56] Moridi M, Masoudi A, Vaez Torshizi R, Hill E (2013). Mitochondrial DNA D‐loop sequence variation in maternal lineages of I ranian native horses. Anim Genet.

[CR57] Nazari F, Seyedabadi H-R, Noshary A, Emamjomeh-Kashan N, Banabazi M-H (2022). A Genome-Wide Scan for Signatures of Selection in Kurdish Horse Breed. J Equine Vet Sci.

[CR58] Newman D, Pilson D (1997). *Increased probability of extinction due to decreased genetic effective population size: experimental populations of Clarkia pulchella*, Vol 51. 10.1111/j.1558-5646.1997.tb02422.x 28565367

[CR59] Norton EM, Avila F, Schultz NE, Mickelson JR, Geor RJ, McCue ME (2019). Evaluation of an HMGA2 variant for pleiotropic effects on height and metabolic traits in ponies. J Vet Intern Med.

[CR60] Palstra FP, Ruzzante DE (2008). Genetic estimates of contemporary effective population size: what can they tell us about the importance of genetic stochasticity for wild population persistence?. Mol Ecol.

[CR61] Petersen JL, Mickelson JR, Cothran EG, Andersson LS, Axelsson J, Bailey E (2013). Genetic diversity in the modern horse illustrated from genome-wide SNP data. PLOS ONE.

[CR62] Pook T, Mayer M, Geibel J, Weigend S, Cavero D, Schoen CC (2020). Improving imputation quality in BEAGLE for crop and livestock data. G3 (Bethesda, Md).

[CR63] Purcell S, Neale B, Todd-Brown K, Thomas L, Ferreira MA, Bender D (2007). PLINK: a tool set for whole-genome association and population-based linkage analyses. Am J Hum Genet.

[CR64] Quan J, Ding R, Wang X, Yang M, Yang Y, Zheng E (2018). Genome-wide association study reveals genetic loci and candidate genes for average daily gain in Duroc pigs. Asian-Australas J Anim Sci.

[CR65] R Core Team (2013). R: A language and environment for statistical computing.

[CR66] Rahimi-Mianji G, Nejati-Javaremi A, Farhadi A (2015). Genetic diversity, parentage verification, and genetic bottlenecks evaluation in iranian turkmen horse1. Russian J Genet.

[CR67] Rajawat D, Panigrahi M, Kumar H, Nayak SS, Parida S, Bhushan B (2022). Identification of important genomic footprints using eight different selection signature statistics in domestic cattle breeds. Gene.

[CR68] Randhawa IAS, Khatkar MS, Thomson PC, Raadsma HW (2014). Composite selection signals can localize the trait specific genomic regions in multi-breed populations of cattle and sheep. BMC Genet.

[CR69] Remer V, Bozlak E, Felkel S, Radovic L, Rigler D, Grilz-Seger G (2022). Y-Chromosomal Insights into Breeding History and Sire Line Genealogies of Arabian Horses. Genes (Basel).

[CR70] Reynolds J, Weir BS, Cockerham CC (1983). Estimation of the coancestry coefficient: basis for a short-term genetic distance. Genetics.

[CR71] Rimbault M, Beale HC, Schoenebeck JJ, Hoopes BC, Allen JJ, Kilroy-Glynn P (2013). Derived variants at six genes explain nearly half of size reduction in dog breeds. Genome Res.

[CR72] Sabeti PC, Varilly P, Fry B, Lohmueller J, Hostetter E, Cotsapas C (2007). Genome-wide detection and characterization of positive selection in human populations. Nature.

[CR73] Sadeghi R, Moradi-Shahrbabak M, Miraei Ashtiani SR, Schlamp F, Cosgrove EJ, Antczak DF (2019). Genetic diversity of Persian Arabian Horses and their relationship to other native Iranian horse breeds. J Heredity.

[CR74] Sakaue S, Kanai M, Tanigawa Y, Karjalainen J, Kurki M, Koshiba S (2021). A cross-population atlas of genetic associations for 220 human phenotypes. Nat Genet.

[CR75] Salek AS, Zandi MB, Vahedi SM, Janssens S (2022). Population structure and genomic footprints of selection in five major Iranian horse breeds. Anim Genet.

[CR76] Saravanan KA, Panigrahi M, Kumar H, Bhushan B (2022). Advanced software programs for the analysis of genetic diversity in livestock genomics: a mini review. Biol Rhythm Res.

[CR77] Saravanan KA, Panigrahi M, Kumar H, Bhushan B, Dutt T, Mishra BP (2021). Genome-wide analysis of genetic diversity and selection signatures in three Indian sheep breeds. Livest Sci.

[CR78] Schlamp F, van der Made J, Stambler R, Chesebrough L, Boyko AR, Messer PW (2016). Evaluating the performance of selection scans to detect selective sweeps in domestic dogs. Mol Ecol.

[CR79] Seyedabadi H, Amirinia S, Bana BM, Emrani H (2006). Parentage verification of Iranian Caspian horse using microsatellites markers.

[CR80] Shahsavarani H, Rahimi-Mianji G (2010). Analysis of genetic diversity and estimation of inbreeding coefficient within Caspian horse population using microsatellite markers. Afr J Biotechnol.

[CR81] Slynko I, Nguyen S, Hamilton EMC, Wisse LE, de Esch IJP, de Graaf C (2021). Vanishing white matter: Eukaryotic initiation factor 2B model and the impact of missense mutations. Mol Genet Genom Med.

[CR82] Smith K, McCoy KD, Macpherson AJ (2007). Use of axenic animals in studying the adaptation of mammals to their commensal intestinal microbiota. Semin Immunol.

[CR83] Sun M-S, Zhang J, Jiang L-Q, Pan Y-X, Tan J-Y, Yu F (2018). TMED2 Potentiates Cellular IFN Responses to DNA Viruses by Reinforcing MITA Dimerization and Facilitating Its Trafficking. Cell Rep..

[CR84] Takezaki N, Nei M (1996). Genetic distances and reconstruction of phylogenetic trees from microsatellite DNA. Genetics.

[CR85] Todorov V, Templ M, Filzmoser P (2011). Detection of multivariate outliers in business survey data with incomplete information. Adv Data Anal Classification.

[CR86] VanRaden PM, Olson KM, Wiggans GR, Cole JB, Tooker ME (2011). Genomic inbreeding and relationships among Holsteins, Jerseys, and Brown Swiss. J Dairy Sci.

[CR87] Vatsiou AI, Bazin E, Gaggiotti OE (2016). Detection of selective sweeps in structured populations: a comparison of recent methods. Mol Ecol.

[CR88] Venables WN, Ripley BD (2013). *Modern applied statistics with S-PLUS*. Springer Science & Business Media.

[CR89] Verity R, Collins C, Card DC, Schaal SM, Wang L, Lotterhos KE (2017). minotaur: A platform for the analysis and visualization of multivariate results from genome scans with R Shiny. Mol Ecol Resour.

[CR90] Wallner B, Palmieri N, Vogl C, Rigler D, Bozlak E, Druml T (2017). Y Chromosome Uncovers the Recent Oriental Origin of Modern Stallions. Curr Biol.

[CR91] Waples RS, Do C (2008). ldne: a program for estimating effective population size from data on linkage disequilibrium. Mol Ecol Resour.

[CR92] Wright S (1931). Evolution in Mendelian populations. Genetics.

[CR93] Wright S (1949). The genetical structure of populations. Ann Eugen.

[CR94] Wu T, Hu E, Xu S, Chen M, Guo P, Dai Z (2021). clusterProfiler 4.0: A universal enrichment tool for interpreting omics data. Innovation.

[CR95] Yousefi-Mashouf N, Mehrabani-Yeganeh H, Nejati-Javaremi A, Bailey E, Petersen JL (2021). Genomic comparisons of Persian Kurdish, Persian Arabian and American Thoroughbred horse populations. PLOS ONE.

[CR96] Yurchenko AA, Daetwyler HD, Yudin N, Schnabel RD, Vander Jagt CJ, Soloshenko V (2018). Scans for signatures of selection in Russian cattle breed genomes reveal new candidate genes for environmental adaptation and acclimation. Sci Rep..

[CR97] Zheng X, Levine D, Shen J, Gogarten SM, Laurie C, Weir BS (2012). A high-performance computing toolset for relatedness and principal component analysis of SNP data. Bioinformatics.

