# Supplementary material for: Genetic diversity and signatures of selection in four indigenous horse breeds of Iran
Source: Heredity (Edinb). 2023 Jun 12;131(2):96–108. doi: 10.1038/s41437-023-00624-7 (PMC10382556; doi:10.1038/s41437-023-00624-7)
Supplement: Supplementary file 1 — Supplementary file [file 41437_2023_624_MOESM1_ESM.docx]

**Table S1.** Description of the datasets used in this study.

| **Data set** | **Genotyping Array** | **Breed** | **No. samples (male)** |
| --- | --- | --- | --- |
| Sadeghi et al., 2019 | Axiom Equine 670K | Turkemen | 11 (5) |
|  |  | Caspian | 7 (4) |
|  |  | Kurdish | 7 (4) |
|  |  | Persian Arabians | 71 (23) |
| Cosgrove et al., 2020 | Axiom Equine 670K | Persian Arabians | 9 (2) |
| National Animal Breeding Center (ABC) | GGP Equine 70K | Turkemen | 34 (30) |
|  |  | Caspian | 22 (7) |
|  |  | Kurdish | 30 (28) |
| Animal Science Research Institute of IRAN (ASRI) | GGP Equine 70K | Turkemen | 2 (1) |
|  |  | Kurdish | 81 (51) |
|  |  | Persian Arabians | 1 (1) |
| Total | | | 275 |

**Table S2**. Information about the investigated horse breeds.

| **Breed** | **Origin (province)** | **Population in Iran^1^** | **Characteristics** |
| --- | --- | --- | --- |
| Caspian | North of Iran (Mazandaran and Gilan) | 1006 | Short height; Talented for riding and excellent for training to children; They have a slim body with a graceful neck, sloping shoulders, straight back; For more details see [Hosseini *et al.* (2016)](#_ENREF_2) |
| Turkmen | Russia, Turkmenistan and the North of Iran (Golestan and North Khorasan) | 5797 | Medium height; Talented for jumping and course performances; they are an endurance horse with a slender body; Having silky body surface hair with special luminosity; For more details see [Hosseini *et al.* (2016)](#_ENREF_2) and [Ghezelsoflou *et al.* (2018)](#_ENREF_1) |
| Kurdish | West of Iran (Kurdistan) | 7640 | Medium height; Talented for polo and dressage; very capable in mountain hiking; Bulged forehead, muscular body, short and strong legs and feet; for more details see [Yousefi Mashouf *et al.* (2020)](#_ENREF_4) and [Hosseini *et al.* (2016)](#_ENREF_2) |
| Persian Arabian | Southwest of Iran (Khuzestan) | 13080 | Medium height; Talented for course performance; They have a smooth forehead with an elongated and muscular body, and a very strong back; For more information about body characteristics see [Mostafavi *et al.* (2019)](#_ENREF_3) |

^1^ Data were retrieved from the National Animal Breeding Center (ABC) of Iran in 2019.

**Table S3**. The average of monthly climate data (standard deviation) for the geographic regions of origin for the four Iranian horse populations for the years 1970-2000. This information was retrieved from the WorldClim version 2.1 climate database (<https://www.worldclim.org/>).

| **Breed** | **Origin in Iran** | **Annual mean temperature** | **Max temperature of warmest month** | **Min temperature of coldest month** | **Annual precipitation** | **Average altitude** |
| --- | --- | --- | --- | --- | --- | --- |
| Turkmen | Golestan/North Khorasan | 14.6 (3.02) | 33.4 (1.78) | -2.6 (4.51) | 27.8 (6.19) | 723.3 |
| Caspian | Mazandaran/Gilan | 14 (2.97) | 30.3 (1.18) | 0 (4.88) | 74 (31.83) | 679.4 |
| Kurdish | Kurdistan | 10.5 (1.46) | 32.9 (1.45) | -7.7 (1.56) | 50 (10.12) | 1869.5 |
| Persian Arabian | Khuzestan | 24.5 (1.57) | 44.5 (1.52) | 6.4 (1.87) | 27.1 (8.31) | 145.1 |

**Table S4**. Information about the outliers detected based on the first three PCs using Local outlier factor (LOF) algorithm.

| **Breed** | **Dataset** | **No. of outlier (male)** |
| --- | --- | --- |
| Caspian | ABC | 1 (0) |
| Turkmen | ABC | 3 (2) |
| Kurdish | ASRI | 4 (2) |
|  | ABC | 7 (5) |
| Persian Arabian | Sadeghi et al., 2019 | 4 (0) |

**Table S5.** Estimates of Runs of Homozygosity (ROH) for each breed using five different length categories.

| **Length category** | **Persian Arabian** | **Caspian** | **Kurdish** | **Turkmen** |
| --- | --- | --- | --- | --- |
| 0-4 Mb | 18136 | 7248 | 23739 | 9634 |
| 4-8 Mb | 572 | 68 | 353 | 131 |
| 8-16 Mb | 166 | 48 | 68 | 51 |
| 16-32 Mb | 76 | 29 | 21 | 24 |
| >32 Mb | 9 | 16 | 13 | 6 |
| Sum | 18959 | 7409 | 24194 | 9846 |

**Table S6.** Position of ROHs that were shared in more than 70% of the horses within each population.

| **Breed** | **Start SNP** | **End SNP** | **Chr (start; length)** | **nSNP** |
| --- | --- | --- | --- | --- |
| Turkmen | MNEc.2.10.24531877.TBIEC2-116258 | MNEc.2.10.25515847.BIEC2-116627 | 10 (24965648; 986516) | 18 |
| Turkmen | MNEc.2.14.59493316.BIEC2-260079 | MNEc.2.14.60346562.BIEC2-260188 | 14 (58823026; 1074820) | 12 |
| Caspian | MNEc.2.1.34004616.TBIEC2-16253 | MNEc.2.1.34320315.BIEC2-15499 | 1 (34176679; 315785) | 7 |
| Caspian | MNEc.2.10.24531877.TBIEC2-116258 | MNEc.2.10.24808945.BIEC2-116319 | 10 (24965648; 280792) | 7 |
| Caspian | MNEc.2.11.33969533.BIEC2-149920 | MNEc.2.11.34022281.TBIEC2-157657 | 11 (34267072; 52741) | 3 |
| Caspian | MNEc.2.11.36847420.BIEC2-150506 | MNEc.2.11.37260015.BIEC2-150682 | 11 (37142586; 413011) | 7 |
| Caspian | MNEc.2.14.59493316.BIEC2-260079 | MNEc.2.14.59873032.BIEC2-260142 | 14 (58823026; 601392) | 4 |
| Caspian | MNEc.2.14.60137821.BIEC2-260166 | MNEc.2.14.60221024.BIEC2-260173 | 14 (59689196; 83206) | 3 |
| Caspian | MNEc.2.21.10002112.BIEC2-550941 | MNEc.2.21.10543538.BIEC2-551251 | 21 (10705149; 540726) | 18 |
| Caspian | MNEc.2.23.52629401.BIEC2-627717 | MNEc.2.23.52629401.BIEC2-627717 | 23 (52438939; 1) | 1 |
| Caspian | MNEc.2.24.2944087.BIEC2-629444 | MNEc.2.24.2954165.BIEC2-629450 | 24 (2809115; 10078) | 2 |
| Caspian | MNEc.2.24.3323322.BIEC2-629493 | MNEc.2.24.3816426.BIEC2-629630 | 24 (3188208; 492909) | 9 |
| Caspian | MNEc.2.28.17547381.BIEC2-732240 | MNEc.2.28.17630541.BIEC2-732339 | 28 (18581459; 83137) | 5 |
| Caspian | MNEc.2.3.19281203.BIEC2-773762 | MNEc.2.3.19935347.BIEC2-813438 | 3 (19974289; 654085) | 15 |
| Caspian | MNEc.2.5.48267461.BIEC2-908026 | MNEc.2.5.49689358.BIEC2-955929 | 5 (44816748; 1449285) | 15 |
| Caspian | MNEc.2.7.54273371.BIEC2-1000611 | MNEc.2.7.54766143.TBIEC2-1055606 | 7 (56285871; 492593) | 7 |
| Persian Arabian | MNEc.2.10.24763315.BIEC2-110900 | MNEc.2.10.25515847.BIEC2-116627 | 10 (25200902; 751262) | 13 |
| Persian Arabian | MNEc.2.16.69542250.BIEC2-357595 | MNEc.2.16.70180289.BIEC2-357998 | 16 (71123941; 640015) | 16 |
| Persian Arabian | MNEc.2.17.80218436.BIEC2-387812 | MNEc.2.17.80522744.BIEC2-387976 | 17 (80146011; 342014) | 7 |
| Persian Arabian | MNEc.2.18.29608306.BIEC2-431061 | MNEc.2.18.29681659.BIEC2-409966 | 18 (29639520; 73353) | 2 |
| Persian Arabian | MNEc.2.18.29763701.BIEC2-409977 | MNEc.2.18.29763701.BIEC2-409977 | 18 (29794915; 1) | 1 |
| Persian Arabian | MNEc.2.3.34487758.BIEC2-776635 | MNEc.2.3.34499919.BIEC2-776644 | 3 (35179337; 12161) | 4 |
| Persian Arabian | MNEc.2.4.87204839.BIEC2-872849 | MNEc.2.4.87204839.BIEC2-872849 | 4 (87320250; 1) | 1 |
| Persian Arabian | MNEc.2.9.31682132.BIEC2-1085345 | MNEc.2.9.32111050.TBIEC2-1145214 | 9 (32537122; 429449) | 5 |

**Table S7.** SNPs and their nearby genes and QTLs as candidates for selective signals using the DCMS test on 31 horse autosomes for the six possible pairwise comparisons.

| **Pairwise comparison** | **SNP (chr: position)** | **DCMS** | **q-values** | **Gene stable ID (symbol)** | **QTL type (QTL ID)^1^** | **Associated with height in human (no. of studies)** |
| --- | --- | --- | --- | --- | --- | --- |
| Turkmen vs Caspian | MNEc.2.1.143354555.BIEC2-62658 (1: 144822337) | 11.58 | 0.0483 | - | chronic progressive lymphedema QTL (28316; 28297); Osteochondrosis QTL (27159) | - |
|  | MNEc.2.6.81216957.BIEC2-1024137 (6: 82381490) | 13.91 | 0.0027 | ENSECAG00000035175; ENSECAG00000034317; ENSECAG00000041572 | - | - |
|  | MNEc.2.6.81451782.BIEC2-1024200 (6: 82615517) | 14.59 | 0.0015 | ENSECAG00000040252 (HMGA2); ENSECAG00000026338 (eca-mir-763) | - | HMGA2 (22) |
|  | MNEc.2.6.81557294.BIEC2-1024233 (6: 82721096) | 12.7 | 0.0142 | - | - | - |
|  | MNEc.2.6.81643314.BIEC2-971389 (6: 82807129) | 11.71 | 0.0443 | ENSECAG00000006721; ENSECAG00000005815 (LLPH) | Alternate gaits QTL (172487; 172495); Withers height QTL (165888) | LLPH (2) |
|  | MNEc.2.6.81643501.BIEC2-971390 (6: 82807316) | 15.15 | 0.001 | ENSECAG00000006721; ENSECAG00000005815 (LLPH) | Alternate gaits QTL (172487*; 172495*); Withers height QTL (165888*) | LLPH (2) |
|  | MNEc.2.8.34988337.BIEC2-1043651 (8: 38017096) | 12 | 0.0303 | - | - | - |
|  | MNEc.2.8.35025281.BIEC2-1043683 (8: 38053942) | 12.93 | 0.0127 | - | - | - |
|  | MNEc.2.10.30642716.BIEC2-112411 (10: 31239758) | 12.67 | 0.0142 | ENSECAG00000019440 (IMPG1) | - | - |
|  | MNEc.2.11.31922134.BIEC2-149102 (11: 32220976) | 11.55 | 0.0483 | ENSECAG00000011435 (MSI2) | - | MSI2 (2) |
|  | MNEc.2.14.15169364.BIEC2-245869 (14: 14402678) | 13.91 | 0.0027 | - | - | - |
|  | MNEc.2.16.40673136.BIEC2-342810 (16: 42256149) | 12.45 | 0.0181 | ENSECAG00000002984 (XCR1); ENSECAG00000013718 (FYCO1); ENSECAG00000003105 (CXCR6) | - | - |
|  | MNEc.2.16.40704545.BIEC2-342840 (16: 42287571) | 12.04 | 0.0303 | ENSECAG00000013718 (FYCO1); ENSECAG00000003105 (CXCR6) | - | - |
| Turkmen vs Kurdish | MNEc.2.8.23472519.BIEC2-1036966 (8: 25935415) | 12.77 | 0.0075 | ENSECAG00000006191 (RILPL1); ENSECAG00000034285 (TMED2); ENSECAG00000011237 (DDX55); ENSECAG00000021362 (EIF2B1); ENSECAG00000023959 (GTF2H3) | - | TMED2 (1); DDX55 (1); GTF2H3 (1) |
|  | MNEc.2.8.23619961.BIEC2-1037063 (8: 26082778) | 10.86 | 0.0491 | ENSECAG00000024587 (ATP6V0A2); ENSECAG00000008535 (DNAH10) | - | DNAH10 (1) |
|  | MNEc.2.8.23699131.BIEC2-1037180 (8: 26163583) | 13 | 0.0075 | ENSECAG00000008535 (DNAH10); ENSECAG00000021239 (CCDC92) | - | DNAH10 (1) |
|  | MNEc.2.8.34988337.BIEC2-1043651 (8: 38017096) | 12.26 | 0.0125 | - | - | - |
|  | MNEc.2.8.35025281.BIEC2-1043683 (8: 38053942) | 12.28 | 0.0125 | - | - | - |
|  | MNEc.2.8.35030511.BIEC2-1043694 (8: 38059178) | 11.7 | 0.02 | - | - | - |
|  | MNEc.2.8.35100261.BIEC2-1043785 (8: 38128920) | 12.1 | 0.0125 | - | - | - |
|  | MNEc.2.10.30595382.TBIEC2-117849 (10: 31192416) | 12.18 | 0.0125 | ENSECAG00000024053 (MYO6); ENSECAG00000019440 (IMPG1) | - | MYO6 (1) |
|  | MNEc.2.10.30642716.BIEC2-112411 (10: 31239758) | 12.12 | 0.0125 | ENSECAG00000019440 (IMPG1) | - | - |
|  | MNEc.2.10.30764949.BIEC2-112446 (10: 31361566) | 11.36 | 0.031 | - | - | - |
|  | MNEc.2.10.30804423.BIEC2-112467 (10: 31401040) | 13.43 | 0.0075 | - | - | - |
|  | MNEc.2.10.30864192.BIEC2-117944 (10: 31460844) | 11.13 | 0.0339 | - | - | - |
|  | MNEc.2.10.31734972.BIEC2-112959 (10: 32332656) | 11.12 | 0.0339 | - | - | - |
|  | MNEc.2.10.32064525.BIEC2-113117 (10: 32662170) | 13.98 | 0.0052 | ENSECAG00000007058 (MEI4) | Navicular bone morphology QTL (28450*; 28374*) | - |
|  | MNEc.2.13.28590293.BIEC2-238283 (13: 29752054) | 12.9 | 0.0075 | ENSECAG00000006315 (XYLT1) | - | XYLT1 (3) |
|  | MNEc.2.14.76753610.BIEC2-267205 (14: 76772476) | 11.16 | 0.0339 | ENSECAG00000042329; ENSECAG00000021057 (ADGRV1) | - | ADGRV1 (2) |
|  | MNEc.2.15.75293369.BIEC2-320314 (15: 76561053) | 11.25 | 0.033 | - | - | - |
|  | MNEc.2.15.75957967.BIEC2-320587 (15: 77228257) | 11.31 | 0.0318 | ENSECAG00000036887 | - | - |
|  | MNEc.2.17.1621933.BIEC2-385010 (17: 1690467) | 11.41 | 0.0303 | ENSECAG00000038911; ENSECAG00000009365 (IFT88); ENSECAG00000018313 (EEF1AKMT1) | - | - |
|  | MNEc.2.21.25351351.BIEC2-558248 (21: 26056902) | 11.18 | 0.0339 | ENSECAG00000012362 (C7); ENSECAG00000026905 (RPL37); ENSECAG00000026016 (SNORD72); ENSECAG00000022617 (PRKAA1) | - | C7 (14); RPL37 (1) |
|  | MNEc.2.25.32343396.BIEC2-669906 (25: 32967287) | 12.34 | 0.0125 | ENSECAG00000041009; ENSECAG00000041650 | - | - |
|  | MNEc.2.29.5894188.BIEC2-750654 (29: 6912492) | 12.74 | 0.0075 | ENSECAG00000000558 (KIF5B); ENSECAG00000027603 (U6) | - | U6 (16) |
|  | MNEc.2.29.5990493.BIEC2-750672 (29: 7008768) | 12.84 | 0.0075 | ENSECAG00000014289 (ARHGAP12) | - | ARHGAP12 (1) |
|  | MNEc.2.29.6118463.BIEC2-750687 (29: 7136579) | 12.01 | 0.0135 | ENSECAG00000014289 (ARHGAP12) | - | ARHGAP12 (1) |
|  | MNEc.2.31.13765175.BIEC2-880660 (31: 13799706) | 11.9 | 0.0152 | ENSECAG00000035662 | - | - |
| Turkmen vs Persian Arabian | MNEc.2.2.81971837.BIEC2-493028 (2: 82157501) | 10.47 | 0.048 | ENSECAG00000007395 (FHIP1A) | Immunoglobulin E level QTL (28467*) | - |
|  | MNEc.2.5.3969547.BIEC2-888633 (5: 3980039) | 10.5 | 0.048 | ENSECAG00000009395 (POU2F1) | - | - |
|  | MNEc.2.5.53218124.BIEC2-909009 (5: 49794535) | 11.9 | 0.009 | ENSECAG00000021854 (CASQ2); ENSECAG00000008137 (VANGL1) | Osteochondrosis dissecans QTL (28235*); Osteochondrosis QTL (28236*) | - |
|  | MNEc.2.5.70419034.BIEC2-917011 (5: 67306991) | 10.48 | 0.048 | - | - | - |
|  | MNEc.2.5.73173928.BIEC2-918405 (5: 70065173) | 10.57 | 0.048 | ENSECAG00000022964 (CDC7) | - | CDC7 (1) |
|  | MNEc.2.14.76753610.BIEC2-267205 (14: 76772476) | 10.41 | 0.0483 | ENSECAG00000042329; ENSECAG00000021057 (ADGRV1) | - | ADGRV1 (2) |
|  | MNEc.2.16.70028768.BIEC2-357953 (16: 71612515) | 10.89 | 0.0354 | ENSECAG00000010318 (CEP63); ENSECAG00000012650 (KY) | - | CEP63 (3); KY (2) |
|  | MNEc.2.16.70212536.BIEC2-358011 (16: 71796121) | 10.36 | 0.0486 | ENSECAG00000027337 (U6); ENSECAG00000013862 (EPHB1) | - | U6 (16); EPHB1 (1) |
|  | MNEc.2.16.70239088.BIEC2-358020 (16: 71822673) | 11.29 | 0.0235 | ENSECAG00000013862 (EPHB1) | - | EPHB1 (1) |
|  | MNEc.2.16.70243057.BIEC2-358021 (16: 71826643) | 13.08 | 0.0032 | ENSECAG00000013862 (EPHB1) | - | EPHB1 (1) |
|  | MNEc.2.19.56387340.BIEC2-446984 (19: 59248439) | 11.05 | 0.0307 | ENSECAG00000001621 (GPR15); ENSECAG00000020656 (CLDND1); ENSECAG00000031333 (OR5K1) | - | - |
|  | MNEc.2.27.36824424.BIEC2-720878 (27: 37027788) | 12.44 | 0.0041 | ENSECAG00000024680 (CSMD1) | - | CSMD1 (2) |
|  | MNEc.2.27.36824548.BIEC2-720879 (27: 37027912) | 12.44 | 0.0041 | ENSECAG00000024680 (CSMD1) | - | CSMD1 (2) |
| Caspian vs Kurdish | MNEc.2.1.151785528.BIEC2-65046 (1: 153452775) | 11.48 | 0.015 | ENSECAG00000037535 | - | - |
|  | MNEc.2.2.80603809.BIEC2-492286 (2: 80788060) | 10.76 | 0.0323 | ENSECAG00000020605 (TRIM2) | - | TRIM2 (1) |
|  | MNEc.2.2.85137571.BIEC2-494354 (2: 85328820) | 10.61 | 0.0398 | ENSECAG00000016629 (ARHGAP10) | - | - |
|  | MNEc.2.4.85610917.BIEC2-872443 (4: 85725918) | 10.45 | 0.045 | ENSECAG00000024078 (MKLN1) | - | MKLN1 (1) |
|  | MNEc.2.6.81216957.BIEC2-1024137 (6: 82381490) | 12.97 | 0.0044 | ENSECAG00000035175; ENSECAG00000034317; ENSECAG00000041572 | - | - |
|  | MNEc.2.6.81451782.BIEC2-1024200 (6: 82615517) | 12.7 | 0.0048 | ENSECAG00000040252 (HMGA2); ENSECAG00000026338 (eca-mir-763) | - | HMGA2 (22) |
|  | MNEc.2.6.81643314.BIEC2-971389 (6: 82807129) | 11.55 | 0.0141 | ENSECAG00000006721; ENSECAG00000005815 (LLPH) | Withers height QTL (172487; 172495); Alternate gaits QTL (165888) | LLPH (2) |
|  | MNEc.2.6.81643501.BIEC2-971390 (6: 82807316) | 14.86 | 2.00E-04 | ENSECAG00000006721; ENSECAG00000005815 (LLPH) | Alternate gaits QTL (172487*; 172495*); Withers height QTL (165888*) | LLPH (2) |
|  | MNEc.2.7.40906478.BIEC2-996658 (7: 41944626) | 11.29 | 0.0162 | ENSECAG00000009233 (OPCML) | Navicular bone morphology QTL (28408) | OPCML (1) |
|  | MNEc.2.8.23472519.BIEC2-1036966 (8: 25935415) | 11.43 | 0.0153 | ENSECAG00000006191 (RILPL1); ENSECAG00000034285 (TMED2); ENSECAG00000011237 (DDX55); ENSECAG00000021362 (EIF2B1); ENSECAG00000023959 (GTF2H3) | - | TMED2 (1); DDX55 (1); GTF2H3 (1) |
|  | MNEc.2.9.79519370.BIEC2-1106542 (9: 81626613) | 11.98 | 0.0095 | ENSECAG00000024191 (TRAPPC9) | - | - |
|  | MNEc.2.11.19229909.BIEC2-149831 (11: 19292860) | 10.49 | 0.0445 | ENSECAG00000018046 (GRN); ENSECAG00000019870 (ITGA2B); ENSECAG00000017264 (FAM171A2); ENSECAG00000011004 (SLC25A39); ENSECAG00000021953 (RUNDC3A); ENSECAG00000000527 (SLC4A1) | - | GRN (1); SLC25A39 (1); SLC4A1 (1) |
|  | MNEc.2.11.23954865.BIEC2-144514 (11: 24029189) | 11.58 | 0.0141 | ENSECAG00000018160 (TBX21); ENSECAG00000019067 (OSBPL7) | - | TBX21 (1); OSBPL7 (1) |
|  | MNEc.2.11.23955436.BIEC2-144517 (11: 24029760) | 10.44 | 0.045 | ENSECAG00000018160 (TBX21); ENSECAG00000019067 (OSBPL7) | - | TBX21 (1); OSBPL7 (1) |
|  | MNEc.2.11.24017789.BIEC2-144570 (11: 24092051) | 11.3 | 0.0162 | ENSECAG00000019067 (OSBPL7); ENSECAG00000006884 (MRPL10); ENSECAG00000007543 (LRRC46); ENSECAG00000009826 (SCRN2); ENSECAG00000012853 (SP6); ENSECAG00000030150 (eca-mir-9103) | - | OSBPL7 (1) |
|  | MNEc.2.11.24103708.BIEC2-152106 (11: 24177108) | 10.5 | 0.0445 | ENSECAG00000012859 (SP2); ENSECAG00000014995 (PNPO); ENSECAG00000002658 (PRR15L); ENSECAG00000018688 (CDK5RAP3) | Hair density QTL (160243) | SP2 (8); PRR15L (1); CDK5RAP3 (3) |
|  | MNEc.2.11.28957576.BIEC2-147502 (11: 29172299) | 11.61 | 0.0141 | ENSECAG00000033785 | - | - |
|  | MNEc.2.11.29036233.BIEC2-147528 (11: 29250994) | 12.02 | 0.0095 | - | - | - |
|  | MNEc.2.11.29038958.BIEC2-147531 (11: 29253719) | 12.66 | 0.0048 | - | - | - |
|  | MNEc.2.11.29676488.BIEC2-148134 (11: 29937213) | 11.74 | 0.0132 | ENSECAG00000014174 (TOM1L1); ENSECAG00000015531 (COX11) | - | - |
|  | MNEc.2.11.29756053.TBIEC2-155812 (11: 30016785) | 10.8 | 0.0317 | ENSECAG00000016386 (STXBP4) | - | STXBP4 (1) |
|  | MNEc.2.11.29933173.BIEC2-148218 (11: 30193864) | 13.57 | 0.002 | ENSECAG00000040994; ENSECAG00000016953 (HLF) | - | - |
|  | MNEc.2.11.31978508.BIEC2-149127 (11: 32277358) | 11.29 | 0.0162 | ENSECAG00000011435 (MSI2) | Insect bite hypersensitivity QTL (29290; 37892) | MSI2 (2) |
|  | MNEc.2.11.42975749.BIEC2-154341 (11: 43296622) | 12.02 | 0.0095 | ENSECAG00000042180; ENSECAG00000020855 (MYO18A) | Withers height QTL (166105*; 165767*) | MYO18A (1) |
|  | MNEc.2.15.75293369.BIEC2-320314 (15: 76561053) | 12.04 | 0.0095 | - | - | - |
|  | MNEc.2.15.75360632.BIEC2-320329 (15: 76628289) | 11.03 | 0.0241 | - | - | - |
|  | MNEc.2.18.38539113.BIEC2-432432 (18: 38573637) | 10.92 | 0.0276 | ENSECAG00000019682 (ACVR1C) | - | - |
| Caspian vs Persian Arabian | MNEc.2.2.26464809.BIEC2-464675 (2: 26460411) | 10.16 | 0.034 | ENSECAG00000033994; ENSECAG00000043621; ENSECAG00000036712; ENSECAG00000029024 | - | - |
|  | MNEc.2.2.80603809.BIEC2-492286 (2: 80788060) | 10.38 | 0.0319 | ENSECAG00000020605 (TRIM2) | - | TRIM2 (1) |
|  | MNEc.2.2.82035073.BIEC2-493070 (2: 82224419) | 9.94 | 0.0404 | ENSECAG00000007395 (FHIP1A) | Immunoglobulin E level QTL (28467*) | - |
|  | MNEc.2.5.70326926.UKUL1297 (5: 67214673) | 9.85 | 0.0413 | ENSECAG00000021520 (SLC44A3) | - | - |
|  | MNEc.2.6.81216957.BIEC2-1024137 (6: 82381490) | 10.14 | 0.034 | ENSECAG00000035175; ENSECAG00000034317; ENSECAG00000041572 | - | - |
|  | MNEc.2.6.81451782.BIEC2-1024200 (6: 82615517) | 11.69 | 0.0067 | ENSECAG00000040252 (HMGA2); ENSECAG00000026338 (eca-mir-763) | - | HMGA2 (22) |
|  | MNEc.2.6.81643314.BIEC2-971389 (6: 82807129) | 10.44 | 0.0311 | ENSECAG00000006721; ENSECAG00000005815 (LLPH) | Withers height QTL (172487; 172495); Alternate gaits QTL (165888) | LLPH (2) |
|  | MNEc.2.6.81643501.BIEC2-971390 (6: 82807316) | 12.24 | 0.0028 | ENSECAG00000006721; ENSECAG00000005815 (LLPH) | Alternate gaits QTL (172487*; 172495*); Withers height QTL (165888*) | LLPH (2) |
|  | MNEc.2.7.93122365.BIEC2-1072361 (7: 95378767) | 10 | 0.0384 | ENSECAG00000008794 (ANO3); ENSECAG00000010612 (MUC15) | - | - |
|  | MNEc.2.7.93242401.BIEC2-1016804 (7: 95500946) | 10.04 | 0.0382 | ENSECAG00000008794 (ANO3); ENSECAG00000010920 (SLC5A12) | - | SLC5A12 (2) |
|  | MNEc.2.9.32503707.BIEC2-1085779 (9: 33359385) | 10.45 | 0.0311 | ENSECAG00000000087 (SNTG1) | - | - |
|  | MNEc.2.9.32508299.TBIEC2-1145480 (9: 33363988) | 9.87 | 0.0413 | ENSECAG00000000087 (SNTG1) | - | - |
|  | MNEc.2.11.28957576.BIEC2-147502 (11: 29172299) | 9.75 | 0.0452 | ENSECAG00000033785 | - | - |
|  | MNEc.2.11.29036233.BIEC2-147528 (11: 29250994) | 9.75 | 0.0452 | - | - | - |
|  | MNEc.2.11.29038958.BIEC2-147531 (11: 29253719) | 10.19 | 0.034 | - | - | - |
|  | MNEc.2.14.43407303.BIEC2-257386 (14: 42710981) | 10.82 | 0.0217 | ENSECAG00000024817 (P4HA2) | - | P4HA2 (3) |
|  | MNEc.2.15.75293369.BIEC2-320314 (15: 76561053) | 9.89 | 0.0413 | - | - | - |
|  | MNEc.2.15.75360632.BIEC2-320329 (15: 76628289) | 10.14 | 0.034 | - | - | - |
|  | MNEc.2.16.70028768.BIEC2-357953 (16: 71612515) | 10.84 | 0.0217 | ENSECAG00000010318 (CEP63); ENSECAG00000012650 (KY) | - | CEP63 (3); KY (2) |
|  | MNEc.2.16.70180289.BIEC2-357998 (16: 71763956) | 10.29 | 0.034 | ENSECAG00000027337 (U6); ENSECAG00000013862 (EPHB1) | - | U6 (16); EPHB1 (1) |
|  | MNEc.2.16.70239088.BIEC2-358020 (16: 71822673) | 13.21 | 5.00E-04 | ENSECAG00000013862 (EPHB1) | - | EPHB1 (1) |
|  | MNEc.2.16.70243057.BIEC2-358021 (16: 71826643) | 14.59 | 0 | ENSECAG00000013862 (EPHB1) | - | EPHB1 (1) |
|  | MNEc.2.23.22922059.BIEC2-651760 (23: 22313755) | 10.52 | 0.0311 | ENSECAG00000023412 (DMRT3) | Alternate gaits QTL (161283*); Equine sarcoids QTL (28920*); Immunoglobulin G level QTL (28475*) | - |
|  | MNEc.2.24.19344437.BIEC2-670713 (24: 19204360) | 11.07 | 0.0189 | ENSECAG00000009756 (ACOT4); ENSECAG00000016290 (ACOT6); ENSECAG00000033122 (DNAL1) | - | - |
| Kurdish vs Persian Arabian | MNEc.2.1.8158887.BIEC2-2930 (1: 8279684) | 10.04 | 0.0413 | ENSECAG00000023340 (ABRAXAS2) | - | ABRAXAS2 (1) |
|  | MNEc.2.1.13486478.BIEC2-6441 (1: 13602630) | 11.84 | 0.0091 | - | - | - |
|  | MNEc.2.1.102216763.BIEC2-43991 (1: 103128344) | 10.31 | 0.0361 | ENSECAG00000042949 | - | - |
|  | MNEc.2.1.139856731.BIEC2-61431 (1: 140918006) | 10.56 | 0.032 | ENSECAG00000016926 (GABPB1) | - | GABPB1 (1) |
|  | MNEc.2.2.39205199.BIEC2-473937 (2: 39422142) | 10.18 | 0.0361 | ENSECAG00000041880; ENSECAG00000013582 (DHRS3) | Guttural pouch tympany QTL (28265*) | - |
|  | MNEc.2.4.25035459.BIEC2-856021 (4: 24971525) | 10.8 | 0.0241 | ENSECAG00000022762 (LANCL2) | - | - |
|  | MNEc.2.5.70419034.BIEC2-917011 (5: 67306991) | 9.96 | 0.0425 | - | - | - |
|  | MNEc.2.7.86988812.BIEC2-1067243 (7: 89190583) | 10.52 | 0.032 | ENSECAG00000043571; ENSECAG00000039541; ENSECAG00000001949; ENSECAG00000003099; ENSECAG00000030659 | - | - |
|  | MNEc.2.8.49140171.BIEC2-1050293 (8: 52213951) | 12.17 | 0.0063 | ENSECAG00000041607 | Withers height QTL (165928*; 165763*; 165880*) | - |
|  | MNEc.2.8.49140348.BIEC2-1050295 (8: 52214128) | 12.17 | 0.0063 | ENSECAG00000041607 | Withers height QTL (165928*; 165763*; 165880*) | - |
|  | MNEc.2.8.49140411.BIEC2-1050296 (8: 52214191) | 12.17 | 0.0063 | ENSECAG00000041607 | Withers height QTL (165928; 165763; 165880) | - |
|  | MNEc.2.9.27834001.BIEC2-1082719 (9: 28615695) | 11.07 | 0.0201 | ENSECAG00000000568 (TMEM68) | Withers height QTL (166232) | - |
|  | MNEc.2.9.30651153.BIEC2-1084791 (9: 31504693) | 9.93 | 0.0434 | ENSECAG00000040951; ENSECAG00000017579 (RB1CC1) | - | - |
|  | MNEc.2.9.79155548.BIEC2-1106480 (9: 81263217) | 10.98 | 0.0208 | ENSECAG00000024191 (TRAPPC9) | - | - |
|  | MNEc.2.14.60137821.BIEC2-260166 (14: 59689196) | 10.43 | 0.032 | ENSECAG00000043642 (NREP) | - | NREP (1) |
|  | MNEc.2.14.60141889.BIEC2-260170 (14: 59693264) | 10.43 | 0.032 | ENSECAG00000043642 (NREP) | - | NREP (1) |
|  | MNEc.2.14.60221024.BIEC2-260173 (14: 59772402) | 10.43 | 0.032 | ENSECAG00000016183 (STARD4); ENSECAG00000016696 (CAMK4) | - | STARD4 (2) |
|  | MNEc.2.16.27501762.TBIEC2-352650 (16: 28963908) | 11.73 | 0.0091 | ENSECAG00000027644 (eca-mir-1289) | - | - |
|  | MNEc.2.21.34779955.BIEC2-561907 (21: 35802201) | 10.16 | 0.0361 | ENSECAG00000035462 | - | - |
|  | MNEc.2.23.17719021.BIEC2-616222 (23: 17057101) | 10.28 | 0.0361 | ENSECAG00000024312 (RORB) | - | RORB (3) |
|  | MNEc.2.27.36810230.BIEC2-720869 (27: 37013594) | 10.93 | 0.0208 | ENSECAG00000024680 (CSMD1) | - | CSMD1 (2) |
|  | MNEc.2.27.36824424.BIEC2-720878 (27: 37027788) | 11.33 | 0.0148 | ENSECAG00000024680 (CSMD1) | - | CSMD1 (2) |
|  | MNEc.2.27.36824548.BIEC2-720879 (27: 37027912) | 11.3 | 0.0148 | ENSECAG00000024680 (CSMD1) | - | CSMD1 (2) |
|  | MNEc.2.29.21640418.BIEC2-758288 (29: 22723043) | 10 | 0.0417 | ENSECAG00000024146 (UCMA); ENSECAG00000024473 (MCM10); ENSECAG00000007747 (OPTN) | Navicular bone morphology QTL (28411) | - |
|  | MNEc.2.29.21906028.BIEC2-797163 (29: 22987975) | 10.23 | 0.0361 | ENSECAG00000041042; ENSECAG00000019290 (CAMK1D) | Navicular bone morphology QTL (28411*) | CAMK1D (4) |
|  | MNEc.2.29.22096419.BIEC2-758635 (29: 23178305) | 10.23 | 0.0361 | ENSECAG00000019290 (CAMK1D) | - | CAMK1D (4) |
|  | MNEc.2.30.13297937.BIEC2-863515 (30: 13743491) | 10.16 | 0.0361 | ENSECAG00000035899; ENSECAG00000012523 (LYPLAL1) | - | LYPLAL1 (1) |
|  | MNEc.2.30.13887627.BIEC2-821818 (30: 14333480) | 10.03 | 0.0413 | ENSECAG00000041678 (TGFB2) | - | TGFB2 (10) |
| ^1^ QTL IDs with * superscript mean that the respective significant SNP is located within the QTL. | | | | | | |

**Table S8.** Allele frequencies for the Significant SNPs that were common in all the three Caspian’s comparisons identified by the DCMS test.

| **SNP (chr: position)** | **Gene stable ID (symbol)** | **Minor allele** | **Major allele** | **Minor allele frequency** | | | |
| --- | --- | --- | --- | --- | --- | --- | --- |
|  |  |  |  | **Caspian** | **Turkmen** | **Kurdish** | **Arabian** |
| MNEc.2.6.81216957.BIEC2-1024137 (6: 82381490) | ENSECAG00000035175; ENSECAG00000034317; ENSECAG00000041572 | G | A | 0.881 | 0.2414 | 0.2537 | 0.1923 |
| MNEc.2.6.81451782.BIEC2-1024200 (6: 82615517) | ENSECAG00000040252 (HMGA2); ENSECAG00000026338 (eca-mir-763) | C | A | 0.9524 | 0.3276 | 0.3507 | 0.2308 |
| MNEc.2.6.81643501.BIEC2-971390 (6: 82807316) | ENSECAG00000005815 (LLPH); ENSECAG00000006721 | G | A | 0.8571 | 0.2586 | 0.2537 | 0.1635 |
| MNEc.2.6.81643314.BIEC2-971389 (6: 82807129) | ENSECAG00000005815 (LLPH); ENSECAG00000006721 | C | A | 0.8571 | 0.5345 | 0.5 | 0.3077 |

**Table S9.** Significant GO terms for selective signals for the six possible pairwise comparisons.

| **Pairwise comparison** | **ID** | **Description** | **GO domain** | **p-adjust** |
| --- | --- | --- | --- | --- |
| Kurdish vs. Persian Arabian | GO:0004683 | calmodulin-dependent protein kinase activity | Molecular function | 0.0025 |
|  | GO:0031593 | polyubiquitin modification-dependent protein binding | Molecular function | 0.0062 |
| Caspian vs. Persian Arabian | GO:0016790 | thiolester hydrolase activity | Molecular function | 0.001 |
|  | GO:0006637 | acyl-CoA metabolic process | Biological process | 0.0018 |
|  | GO:0006631 | fatty acid metabolic process | Biological process | 0.0249 |
|  | GO:0007548 | sex differentiation | Biological process | 0.0372 |
|  | GO:0060999 | positive regulation of dendritic spine development | Biological process | 0.0372 |
|  | GO:0060996 | dendritic spine development | Biological process | 0.0372 |
|  | GO:0060997 | dendritic spine morphogenesis | Biological process | 0.0372 |
|  | GO:1901214 | regulation of neuron death | Biological process | 0.0372 |
|  | GO:0031290 | retinal ganglion cell axon guidance | Biological process | 0.0372 |
|  | GO:0006629 | lipid metabolic process | Biological process | 0.0372 |
|  | GO:0019226 | transmission of nerve impulse | Biological process | 0.0372 |
|  | GO:0036158 | outer dynein arm assembly | Biological process | 0.0372 |
|  | GO:0046328 | regulation of JNK cascade | Biological process | 0.0372 |
|  | GO:0048593 | camera-type eye morphogenesis | Biological process | 0.0372 |
|  | GO:0031589 | cell-substrate adhesion | Biological process | 0.0372 |
|  | GO:0070372 | regulation of ERK1 and ERK2 cascade | Biological process | 0.0395 |
|  | GO:0007099 | centriole replication | Biological process | 0.0395 |
|  | GO:0007628 | adult walking behavior | Biological process | 0.0395 |
|  | GO:0030010 | establishment of cell polarity | Biological process | 0.0424 |
| Turkmen vs. Persian Arabian | GO:0001618 | virus receptor activity | Molecular function | 0.0269 |
|  | GO:0000979 | RNA polymerase II core promoter sequence-specific DNA binding | Molecular function | 0.0269 |
|  | GO:0005003 | ephrin receptor activity | Molecular function | 0.0269 |
|  | GO:0015026 | coreceptor activity | Molecular function | 0.0269 |
|  | GO:0007194 | negative regulation of adenylate cyclase activity | Biological process | 0.0409 |
|  | GO:0050953 | sensory perception of light stimulus | Biological process | 0.0409 |
|  | GO:0051279 | regulation of release of sequestered calcium ion into cytosol | Biological process | 0.0409 |
|  | GO:0060996 | dendritic spine development | Biological process | 0.0409 |
|  | GO:0060997 | dendritic spine morphogenesis | Biological process | 0.0409 |
|  | GO:1901214 | regulation of neuron death | Biological process | 0.0409 |
|  | GO:0031290 | retinal ganglion cell axon guidance | Biological process | 0.0409 |
|  | GO:0046328 | regulation of JNK cascade | Biological process | 0.0409 |
|  | GO:0048593 | camera-type eye morphogenesis | Biological process | 0.0409 |
|  | GO:0031589 | cell-substrate adhesion | Biological process | 0.0409 |
|  | GO:0070372 | regulation of ERK1 and ERK2 cascade | Biological process | 0.0409 |
|  | GO:0007099 | centriole replication | Biological process | 0.0409 |
|  | GO:0010971 | positive regulation of G2/M transition of mitotic cell cycle | Biological process | 0.0409 |
|  | GO:0002027 | regulation of heart rate | Biological process | 0.0416 |
|  | GO:0030010 | establishment of cell polarity | Biological process | 0.0416 |
|  | GO:0043473 | pigmentation | Biological process | 0.0416 |
|  | GO:0060048 | cardiac muscle contraction | Biological process | 0.0416 |
|  | GO:0046718 | viral entry into host cell | Biological process | 0.0421 |
|  | GO:0045494 | photoreceptor cell maintenance | Biological process | 0.0425 |
|  | GO:0048013 | ephrin receptor signaling pathway | Biological process | 0.0429 |
|  | GO:0033674 | positive regulation of kinase activity | Biological process | 0.0453 |
|  | GO:0050877 | nervous system process | Biological process | 0.0453 |
|  | GO:0022008 | neurogenesis | Biological process | 0.0453 |
|  | GO:0051965 | positive regulation of synapse assembly | Biological process | 0.0453 |
|  | GO:0007154 | cell communication | Biological process | 0.0453 |
| Turkmen vs. Kurdish | GO:0005929 | cilium | Cellular_component | 0.043 |
|  | GO:0042995 | cell projection | Cellular_component | 0.043 |
| Turkmen vs. Caspian | GO:0004950 | chemokine receptor activity | Molecular function | 1.00E-04 |
|  | GO:0006935 | chemotaxis | Biological process | 0.0042 |
|  | GO:0015026 | coreceptor activity | Molecular function | 0.015 |
|  | GO:0019957 | C-C chemokine binding | Molecular function | 0.015 |
|  | GO:0008266 | poly(U) RNA binding | Molecular function | 0.015 |
|  | GO:0016493 | C-C chemokine receptor activity | Molecular function | 0.015 |
|  | GO:0003727 | single-stranded RNA binding | Molecular function | 0.0299 |
|  | GO:0060999 | positive regulation of dendritic spine development | Biological process | 0.0311 |
|  | GO:0003712 | transcription coregulator activity | Molecular function | 0.038 |
|  | GO:0051209 | release of sequestered calcium ion into cytosol | Biological process | 0.0381 |
|  | GO:0034097 | response to cytokine | Biological process | 0.0381 |
|  | GO:0070098 | chemokine-mediated signaling pathway | Biological process | 0.0411 |
|  | GO:0019722 | calcium-mediated signaling | Biological process | 0.0411 |
|  | GO:0090305 | nucleic acid phosphodiester bond hydrolysis | Biological process | 0.0411 |
|  | GO:0060326 | cell chemotaxis | Biological process | 0.0411 |
|  | GO:0007204 | positive regulation of cytosolic calcium ion concentration | Biological process | 0.0411 |
|  | GO:0008201 | heparin binding | Molecular function | 0.0453 |


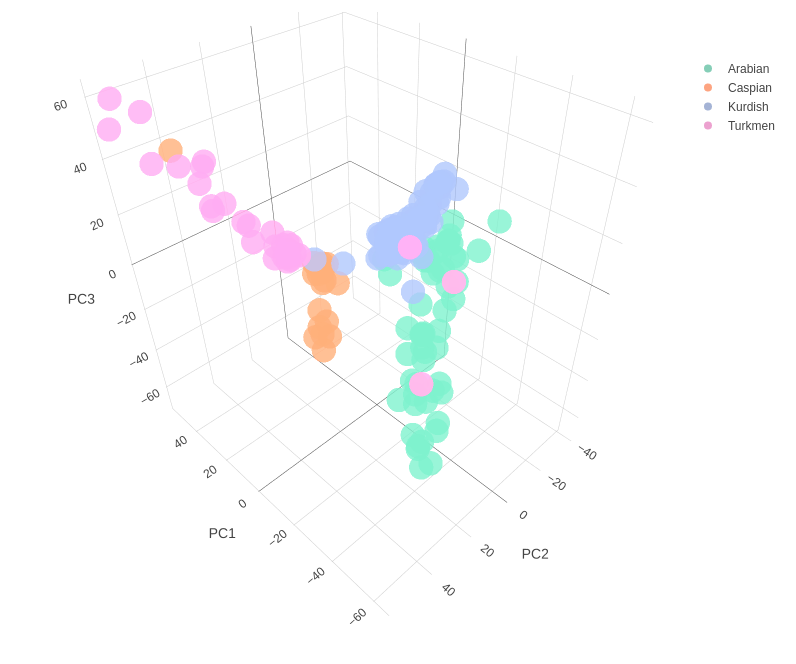


**Figure S1.** 3D PCA scatter plots of the first three principal components (PCs) for the studied breeds before removing outliers.

**
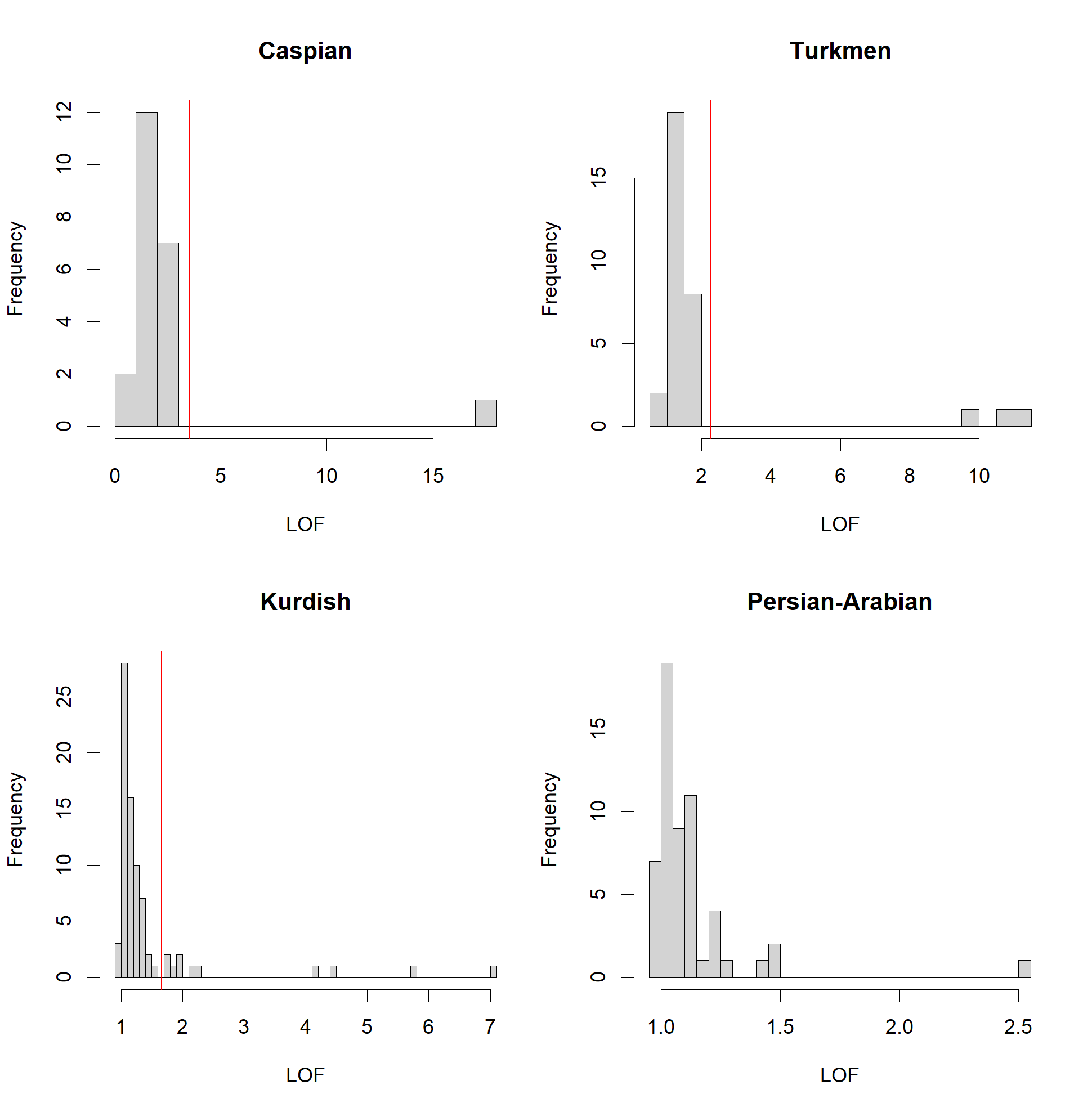
 Figure S2.** Histogram of calculated local outlier factor (LOF) score for each sample in four different breeds. The red vertical line represents the outlier threshold.


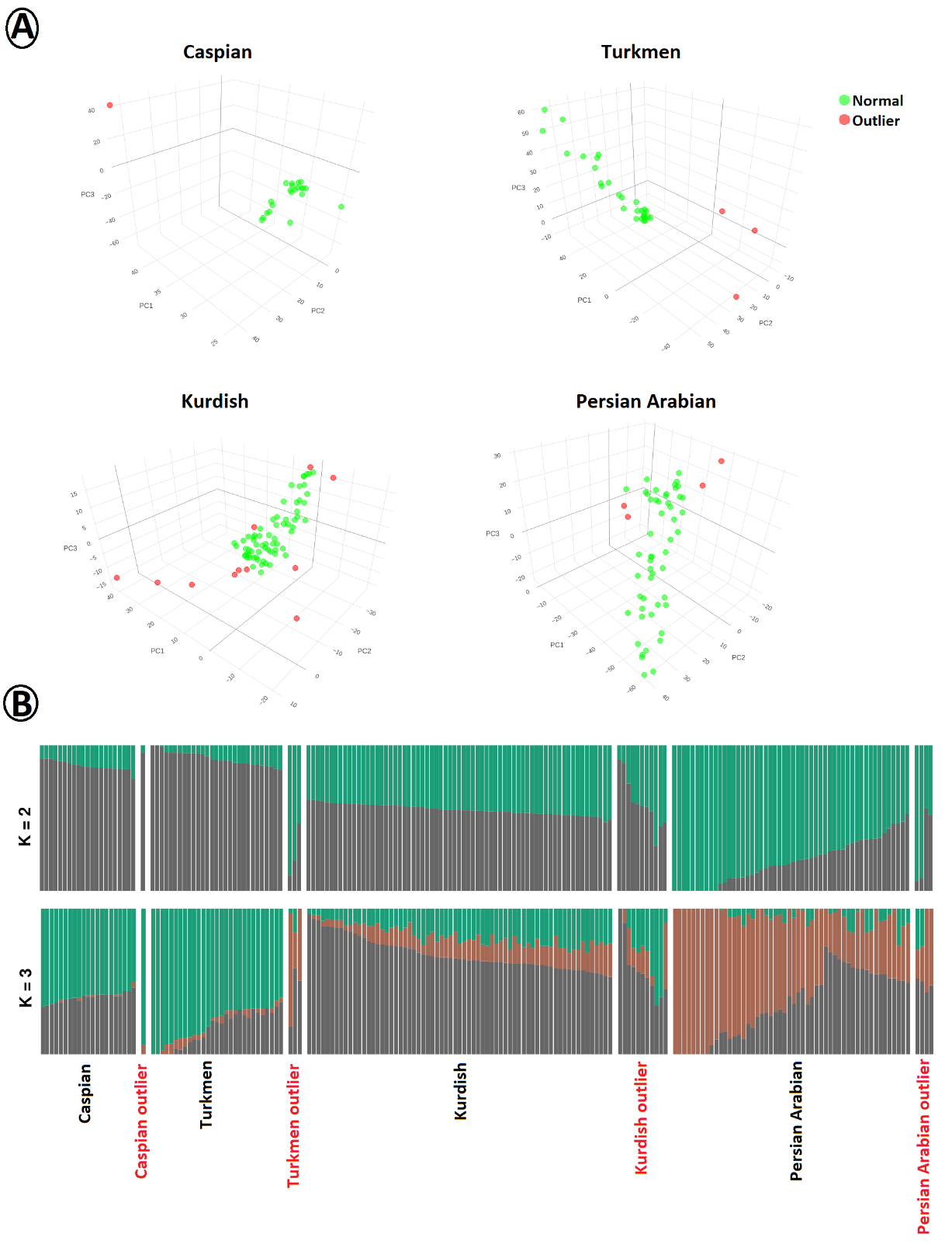


**Figure S3.**  Plots of principle components (A) and Admixture analysis (B) for the detected outliers based on the first three PCs using local outlier factor (LOF) score calculation in four different breeds.


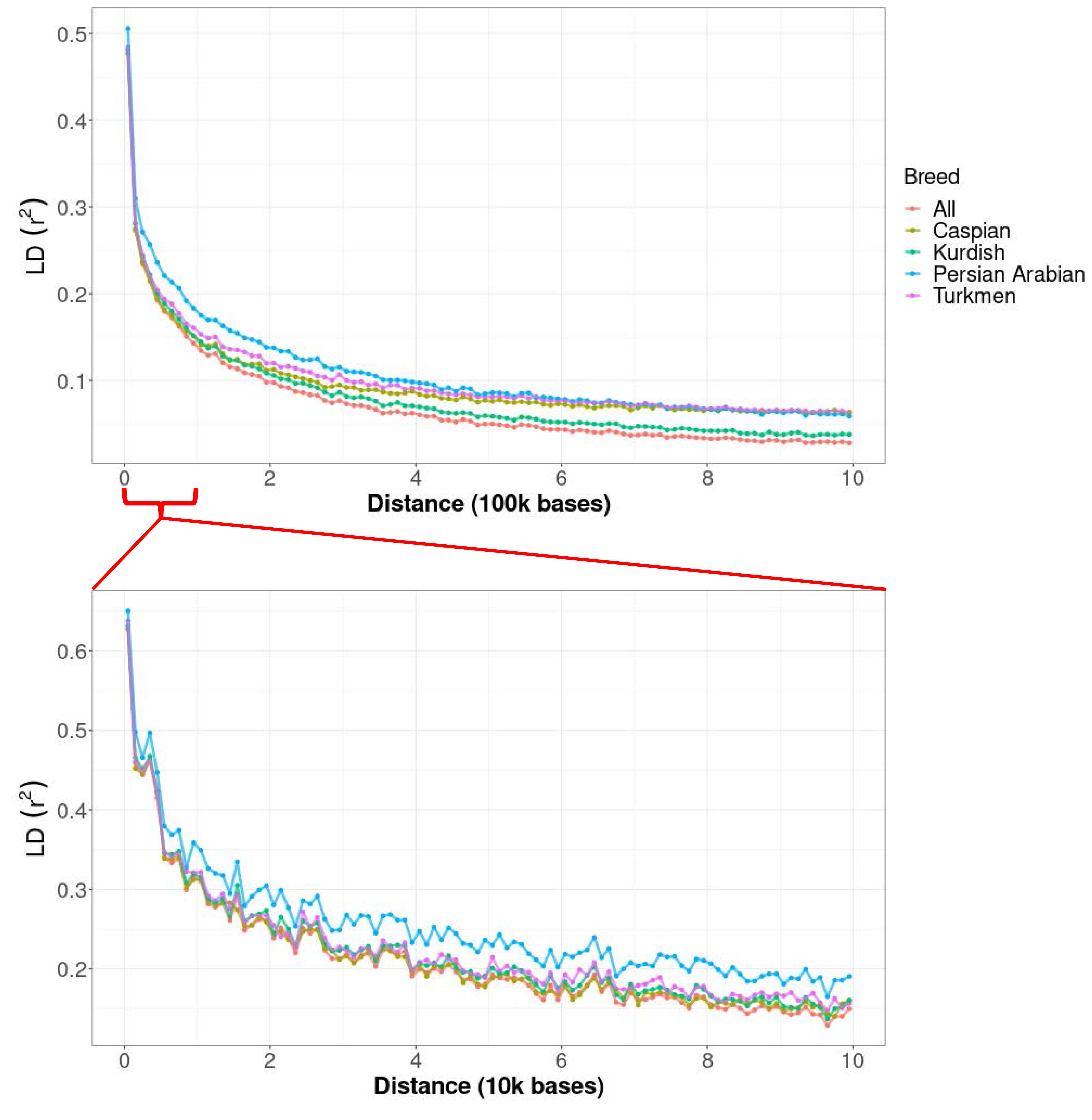


**Figure S4.** Linkage disequilibrium (LD) decay as a function of genomic distance in each population, using r^2^ between pairs of markers in order to understand the pattern of linkage disequilibrium (LD) in the studied breeds.


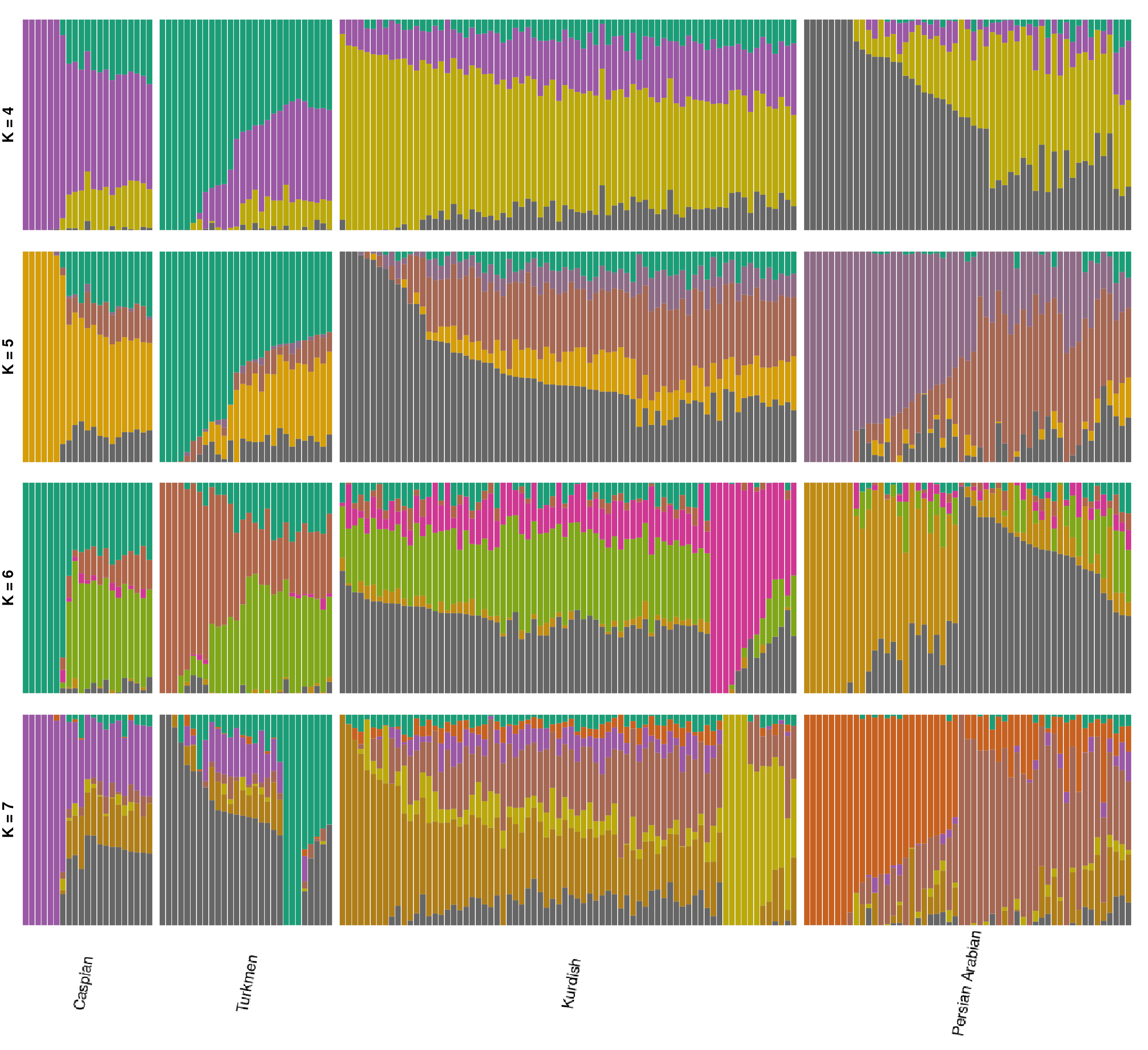


**Figure S5.** Model-based clustering of four Iranian breeds using admixture analysis with the assumed number of ancestries from 4 to 7.


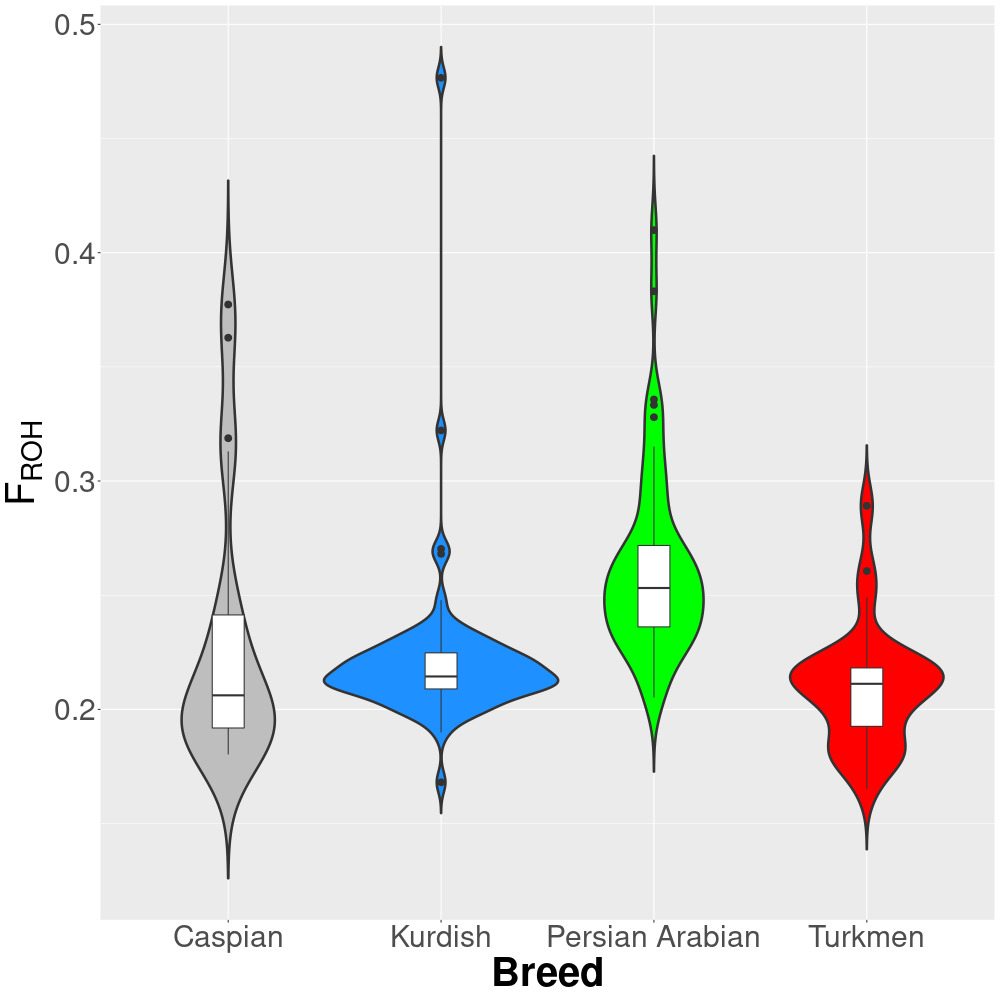


**Figure S6**. The boxplot of inbreeding coefficient base on the length distribution of ROH segments in four studied populations including Turkmen, Caspian, Persian Arabian and Kurdish horses.

**
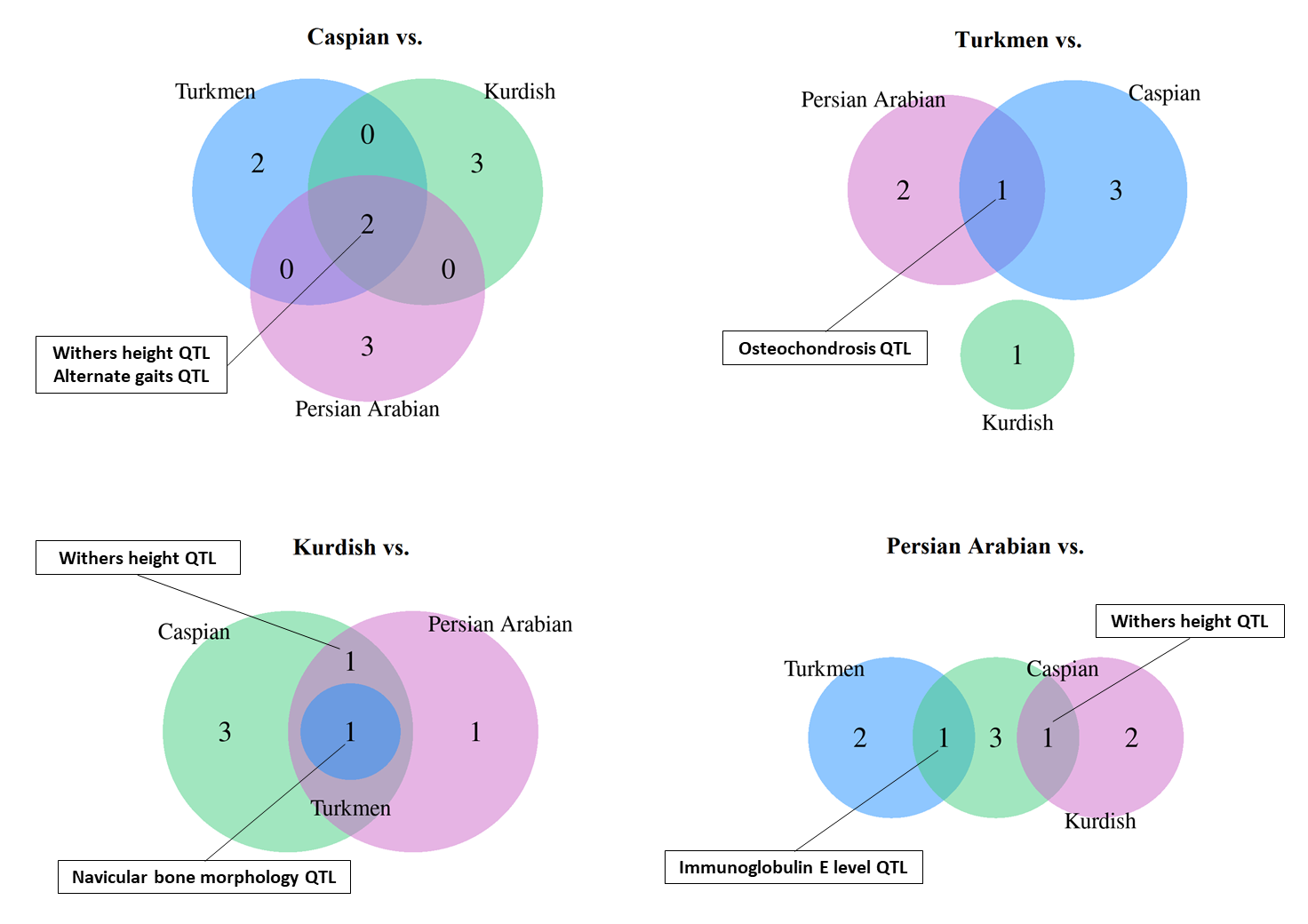
**

**Figure S7.** Venn diagrams summarizing number of overlapped QTL type under selection among different pairwise comparisons.

**
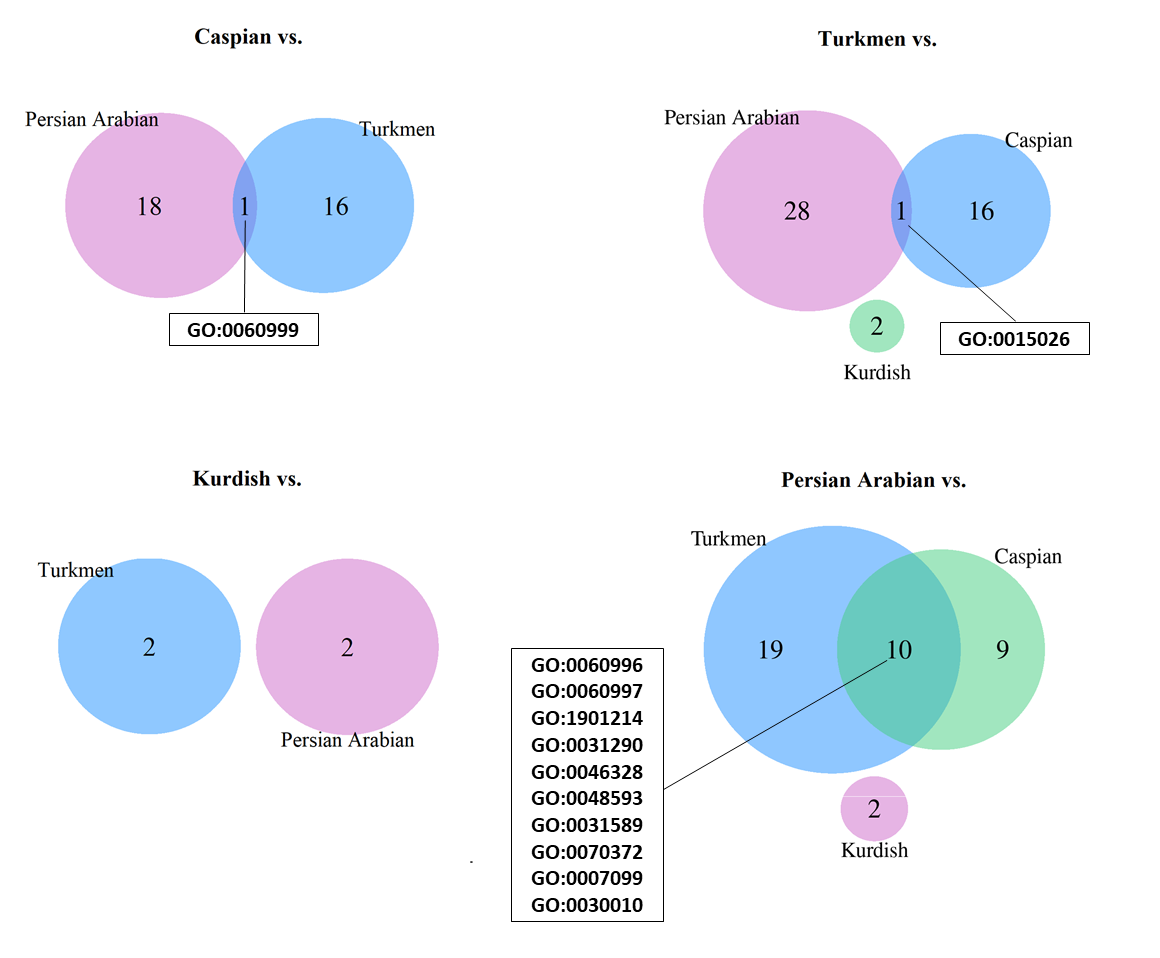
**

**Figure S8.** Venn diagrams summarizing number of overlapped candidate GOs under selection among different pairwise comparisons.

**References:**

Ghezelsoflou, H., P. Hamidi and S. Gharahveysi, 2018 Study of factors affecting the body conformation traits of Iranian Turkoman horses. Journal of equine science 29**:** 91-96.

Hosseini, M., H. M. Shahrbabak, M. B. Zandi and M. Fallahi, 2016 A Morphometric Survey Among Three Iranian Horse Breeds with Multivariate Analysis. Media Peternakan 39**:** 155-160.

Mostafavi, A., M. A. Fozi, A. E. Koshkooieh, M. Mohammadabadi, O. I. Babenko *et al.*, 2019 Effect of LCORL gene polymorphism on body size traits in horse populations. Acta Scientiarum. Animal Sciences 42.

Yousefi Mashouf, N., H. Mehrabani Yeganeh, A. Nejati Javaremi and F. Maloufi, 2020 A Novel Approach to Establish Breed Type and Standards for an Equine Breed: Persian Kurdish Horse. Journal of Agricultural Science and Technology 22**:** 1219-1233.
